# Statistical Parameters Extracted from Radar Sea Clutter Simulated under Different Operational Conditions

**DOI:** 10.3390/s24123720

**Published:** 2024-06-07

**Authors:** Yung-Cheng Pai, Jean-Fu Kiang

**Affiliations:** Graduate Institute of Communication Engineering, National Taiwan University, Taipei 10617, Taiwan

**Keywords:** sea clutter, radar cross-section, statistical parameters, JONSWAP spectrum, Hwang spectrum, Monte Carlo method, sea-surface profile, physical-optics method, probability density function, K distribution, Weibull distribution, power-law distribution, particle swarm optimization, wind speed, wind direction, grazing angle, polarization

## Abstract

A complete framework of predicting the attributes of sea clutter under different operational conditions, specified by wind speed, wind direction, grazing angle, and polarization, is proposed for the first time. This framework is composed of empirical spectra to characterize sea-surface profiles under different wind speeds, the Monte Carlo method to generate realizations of sea-surface profiles, the physical-optics method to compute the normalized radar cross-sections (NRCSs) from individual sea-surface realizations, and regression of NRCS data (sea clutter) with an empirical probability density function (PDF) characterized by a few statistical parameters. JONSWAP and Hwang ocean-wave spectra are adopted to generate realizations of sea-surface profiles at low and high wind speeds, respectively. The probability density functions of NRCSs are regressed with K and Weibull distributions, each characterized by two parameters. The probability density functions in the outlier regions of weak and strong signals are regressed with a power-law distribution, each characterized by an index. The statistical parameters and power-law indices of the K and Weibull distributions are derived for the first time under different operational conditions. The study reveals succinct information of sea clutter that can be used to improve the radar performance in a wide variety of complicated ocean environments. The proposed framework can be used as a reference or guidelines for designing future measurement tasks to enhance the existing empirical models on ocean-wave spectra, normalized radar cross-sections, and so on.

## 1. Introduction

A complete framework of predicting the attributes of sea clutter under specific radar operating conditions is presented for the first time. Field measurement data are not easy to come by. This framework is proposed to predict effectively and efficiently the statistical properties of sea clutter under given conditions of wind and radar by utilizing several state-of-the-art models and methods in different research arenas. This framework is composed of empirical spectra used to characterize sea-surface profiles under different wind speeds, the Monte Carlo method to generate realizations of sea-surface profiles, the physical-optics method to compute the normalized radar cross-sections (NRCSs) from individual sea-surface realizations, and regression of NRCS data (sea clutter) with an empirical probability density function (PDF) characterized by a few statistical parameters. The statistical parameters thus obtained can be used to quickly reproduce the sea clutter under specific operational conditions for radar applications. The proposed framework can be used as a reference or guidelines for designing future measurement tasks to enhance the existing empirical models on ocean-wave spectra, normalized radar cross-sections, and so on. The effectiveness and accuracy of this framework can be further enhanced if the spectra of sea-surface profiles are updated by including more measurement data. In this section, we will review the literature relevant to individual parts of this framework.

Sea clutter can significantly affect the performance of radar surveillance and missile guidance above the sea surface [1,2]. The capricious features of radar sea clutter are affected by the radar parameters like carrier frequency, polarization, and grazing angle, as well as the sea state, which is correlated to the wind speed and direction on the sea surface [3].

Many statistical analyses of measured sea clutter have been presented in the literature, for example, the relation between the radar backscattering coefficient and wind speed [4], the spectra of microwave echoes, and the distribution of sea ripples [5]. However, field measurements were usually constrained by the in situ sea state and the operational radar parameters. A flexible sea-clutter simulator capable of predicting the radar cross-section under various sea states and radar parameters will be very useful in field operations.

For some real-time applications that demand quick response, it will be helpful to have a succinct representation of the sea-clutter distribution in terms of a few statistical parameters, which are contingent upon the sea state and the radar parameters. To achieve this goal, we need proper ocean-wave spectra to simulate sea-surface profiles, proper electromagnetic wave models to compute the radar cross-section from a given sea-surface profile, and proper statistical models to represent the sea clutter. These three constituent parts are reviewed next.

A fully developed sea-surface profile can be characterized by a spectral density function such as the Pierson–Moskowitz (PM) spectrum [6], featuring gravity waves [7], with parameters estimated from the measurement data [8]. However, its effectiveness is less credible in characterizing developing sea surfaces. The JONSWAP spectrum was extended from the Pierson–Moskowitz spectrum and incorporated more wave mechanisms like fetch-limited wave processes [8]. Modified from the JONSWAP spectrum, the V. Yu. Karaev spectrum, T. Elfouhaily spectrum, V. N. Kudryavtsev spectrum, and Hwang spectrum have been used in different scenarios [9].

The directionality embedded in ocean-wave spectra, affected by the wind forcing on the sea surface, has been investigated over decades. An early study of the spectral directionality in [10] was based on field observations in Lake Ontario and a laboratory tank. In [11], several well-established angular spreading functions were reviewed, including cosine type, half-cosine 2*s*-power type, parameterized half-cosine 2*s*-power type, hyperbolic secant-squared type, and composite-structured type. A pattern-sensitive fusion method was proposed to model the sea-surface profile with optimal roughness to account for different ocean environments. The reconstructed random ocean media could be used to compute the electromagnetic scattering from the sea surface. In [12], a Max Planck Institute (MPI) method was applied to estimate the ocean-wave spectrum from acquired synthetic-aperture radar images. The ocean wave spectrum was optimized in terms of a cost function, with the PM spectrum or Elfouhaily spectrum as the initial guess, and the Elfouhaily spectrum turned out to be more suitable as an initial-guess spectrum.

In [13], a geometrical optics small-slope approximation (GO-SSA) model was proposed to compute the radar cross-section (RCS) from sea-surface profiles characterized with a non-directional ocean-wave spectrum at a wind speed of 15 m/s [14]. In [3], an imaging radar systems group (IRSG) was proposed to model the mean backscattering coefficient at low-to-medium grazing angles, and the reflectivity was found to be insensitive to the grazing angle.

In [15], an efficient method for calculating the bistatic scattering from a rough sea surface was proposed. The normalized RCS was computed with the physical-optics (PO) method under a first-order small slope approximation (SSA). Radar polarization and the permittivity of sea water were considered in the physical-optics method, and the SSA was imposed in computing the Kirchhoff integral, which was a surface correlation function of the sea-surface profile. The Kirchhoff integral was related to the probability density function of random surface slope, and the surface correlation function was related to the ocean wave spectrum, thus the normalized RCS was related to the given radar parameters and sea-surface conditions. In [16], an iterative physical-optics method was applied to compute the electromagnetic scattering field, including specular scattering and diffused scattering components, from a sea surface covered with an oil film. The effects of wind speed, oil film thickness, and radar parameters on the scattering field were investigated.

Sea clutter becomes more sensitive to polarization at small grazing angles [2,17]. The radar echo from a rough sea surface at large grazing angles can be modeled by using a Kirchhoff approximation [18]. Predictions under such an approximation matched poorly with real data at small grazing angles due to finite conductivity of the sea water [17]. At low-to-medium grazing angles, the small-perturbation method was applied to model the sea clutter [17]. In a Wright–Valenzuela composite model [19,20], the RCS was given as the Fourier transform of the product of a long-wave component and a short-wave component [17].

A bunch of scatterers at the wave crest may induce multipath reflection [2], which is sensitive to polarization. In [21], vv-dominant scatterers were identified as short-lived slow scatterers and hh-dominant scatterers as long-lived fast scatterers. In [22], the vv backscatter was related to slow scatterers confined to the back side of the sea-surface profile, and the hh backscatter was related to fast scatterers near the sea-surface crest, which became more conspicuous at smaller grazing angles [22].

The attributes of sea clutter at hh and vv polarizations have been reviewed [23]. The backscattered signals at hh polarization were observed less frequently than their counterparts at vv polarization under moderate sea states [24], and the RCS-versus-time curve of the former manifested spiky features [25]. The polarization-related differences were attributed to the fine reflecting facets of wind-driven sea surfaces or small wind ripples [26,27]. vv-polarized backscattering is stronger than hh-polarized backscattering due to local interference induced by capillary waves. On the other hand, spikes were frequently observed in hh polarization with low grazing angles, especially at a high spatial resolution. In a nutshell, Bragg scattering is induced by capillary waves [2] and dominates the vv-polarized echo. Non-Bragg scattering, induced by whitecaps and sea spikes [28], dominates the hh-polarized echo.

Statistical models have been widely used to characterize features of radar sea clutter [29], including mean backscattered power [1,3,13,18], amplitude [17,28,30,31], short-time temporal correlation [32], and the Doppler spectrum [33].

In [13], a small-perturbation two-scale model (TSM) was proposed, by applying a geometric optics (GO) method on long waves and small-slope approximation (SSA) on short waves. At low-to-medium grazing angles, the parameters of the TSM could be well estimated by Bragg scattering [18], being dominated by capillary waves [2]. The results in [2,13,17] suggested that the statistical parameters of sea-clutter distribution vary continuously with the grazing angle.

In [34], the statistical properties of the Doppler velocity derived from sea-spike scattering were investigated and verified with three sets of radar sea-clutter data. The temporal and statistical modeling of scattering from breaking waves agreed well with the measured spikes. In [35], the Doppler spectrum derived from a cliff-top radar experiment was used to verify a sea-clutter model which incorporated Bragg scattering, whitecap scattering, and spikes. The relation between sea-clutter features and wind condition was also studied. In [29], an autoregressive model was proposed to study the modulation on sea clutter attributed to Bragg backscattering induced by long waves. In [36], the temporal variation in Doppler spectra was acquired by fitting the sea clutter to a compound K distribution, considering Bragg and non-Bragg scattering from short and long waves. In [37], the correlation between the mean Doppler shift and the local spectrum intensity was studied under up- and downwind conditions.

Weibull, log-normal, and K distributions have generally been used to characterize sea-clutter data [2,17,30]. In [38], a two-parameter K distribution was developed to fit sea-clutter data affected by capillary waves, wind waves, and gravity waves. It was claimed that a compound model would be useful to characterize the sea clutter attributed to Bragg and non-Bragg scattering [35,36,37,39].

The outlier regions in the sea-clutter distribution may become more conspicuous in high-resolution radar images. Non-Bragg scattering was reported to raise the outlier region of a K distribution [31,35]. In [30], a Pareto distribution was used to better fit the sea clutter in the presence of surface spikes. The shape parameter and scale parameter were successfully estimated by applying a maximum-likelihood estimator on high-resolution radar images.

In [28], a KK distribution was proposed to better fit strong backscattering signals at horizontal polarization. At medium grazing angles, a K+Rayleigh distribution could fit the data better than the K and Pareto+noise distributions [40]. In [28,30,40], the distribution of RCS on high-resolution radar images affected by non-Bragg scattering were well fit with a KK distribution. In [3,41], an Ingara airborne multi-mode X-band radar system was developed to collect fully polarimetric data in a circular spotlight mode, at grazing angles of 10–45°. The data of hh, hv, and vv polarizations were fit with K, KA, and KK distributions, respectively. The KK distribution turned out to fit the data well, including the outlier region attributed to sea spikes [42].

However, the statistical parameters of the compound models, such as shape and scale parameters, are insensitive to the properties of radar echoes under different sea states and radar parameters. The distribution parameters regressed with the measured or simulated RCS data may not be unique due to the complexity of the compound models.

As scattering from the sea surface involves many complicated processes, the sea-surface profile changes with time in a random and complicated manner, which is typically characterized with an empirical ocean-wave spectrum like JONSWAP, with parameters regressed from measurement data. The spectrum represented in the frequency ($\omega_{w}$) domain can be used to describe the time variation in individual sea-surface profiles. The spectrum can also be represented in the spectral (${\bar{k}}_{w}$) domain to reconstruct snapshots of individual sea-surface profiles. A Monte Carlo simulation method can be applied to the spectrum to generate realizations of sea-surface profiles in terms of horizontal position $\bar{r}$ and time *t*.

Given the incident direction (grazing angle), frequency, and polarization of a radar signal, the scattering field from a realization of a sea-surface profile can be computed by applying proper electromagnetic scattering theory, like the physical-optics method in this work. The normalized radar cross-section (NRCS) derived in terms of the scattering field from one sea-surface realization accounts for one incidence of measurement data. An ensemble of NRCS data computed under given wind conditions and radar parameters is compiled to derive a probability density function (PDF) of NRCSs. The PDF is regressed with a K distribution function to estimate a few statistical parameters for characterizing the attributes of sea clutter under the given wind conditions and radar parameters. Power-law distributions are also used to fit very small and very large NRCS data, respectively, leading to power-law indices as additional statistical parameters. Possible relations of these statistical parameters with respect to the wind conditions and radar parameters are explored. These relations can be used to quickly predict the properties of sea clutter under specific wind and radar conditions.

In this work, a complete framework is proposed for the first time to derive the statistical parameters of sea-clutter distribution under variations in wind speed, wind direction, grazing angle, and polarization. The JONSWAP and Hwang spectra are adopted to realize sea-surface profiles under low-to-moderate and high wind speeds, respectively. A multitude of sea-surface profiles are realized by applying a Monte Carlo method upon the specified ocean-wave spectrum. A physical-optics method is applied to compute the normalized radar cross-sections (NRCSs) from individual sea-surface realizations, considering the finite conductivity of the sea water. The NRCS data are compiled to form a probability density function (PDF), which is regressed with the K and Weibull distributions, each characterized by two parameters. The NRCS data with very small and very large amplitudes are also regressed with power-law distributions, each characterized by an index, to explore subtle features under different operational conditions. These statistical parameters of sea clutter are studied under variations in wind speed, wind direction, grazing angle, and polarization.

The rest of this paper is organized as follows. The realization of sea-surface profiles with the JONSWAP and Hwang spectra is presented in Section 2; the computation of a normalized radar cross-section from a given sea-surface profile is presented in Section 3. In Section 4, the PDFs of the NRCSs are regressed with the K and Weibull distributions by using a particle swarm optimization method, and the PDFs in the outlier regions are fit with a power-law distribution by using a weighted linear regression method. The simulation results under systematic variations in wind speed, wind direction, grazing angle, and polarization are presented and elaborated in Section 5. Finally, some conclusions are drawn in Section 6.

## 2. Realization of Sea-Surface Profiles

**Figure 1:**
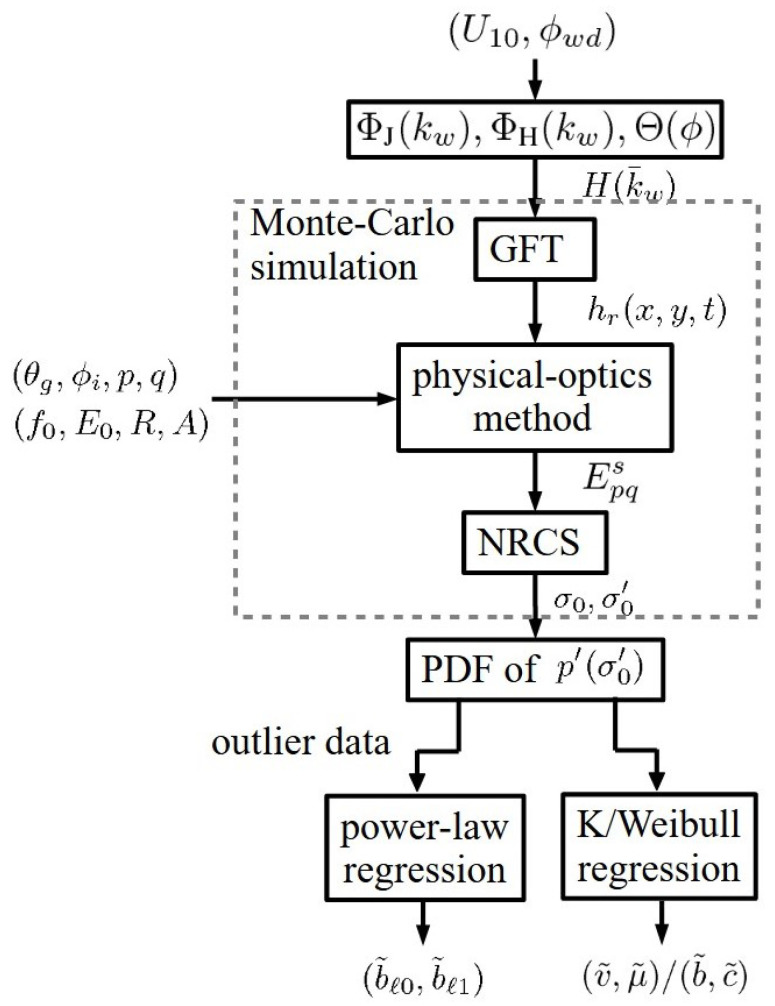
Flowchart of proposed framework.

Figure 1 shows a flowchart of the proposed framework, which is composed of three major parts. The first part constitutes an amplitude spectrum $H({\bar{k}}_{w})$ from either the JONSWAP spectrum $\Phi_{J}\left(\omega_{w}\right)$ or Hwang spectrum $\Phi_{H}\left(\omega_{w}\right)$ under a specific wind speed $U_{10}$ and wind direction $\phi_{wd}$, as well as an associated azimuthal pattern Θ(ϕ).

The second part invokes a Monte Carlo method to realize multiple sea-surface profiles $h_{r}\left(x,y,t\right)$ by applying a generalized Fourier transform (GFT) on the amplitude spectrum $H({\bar{k}}_{w})$. Then, the backscattered electric field $E_{pq}^{s}$ is computed from each sea-surface realization with the physical-optics method under the specified radar parameters of grazing angle $\theta_{g}$, incident azimuth angle $\phi_{i}$, scattering polarization *p*, incident polarization *q*, frequency $f_{0}$, field amplitude $E_{0}$, range *R*, and target area *A*. The $E_{pq}^{s}$ is transformed to the normalized radar cross-section (NRCS) $\sigma_{0}$ in natural units and $\sigma_{0}^{\prime }$ in dB.

In the third part, a probability density function (PDF) $p^{\prime }\left(\sigma_{0}^{\prime }\right)$ is derived from the histogram of $\sigma_{0}^{\prime }$ simulated in the second part. The PDF is then regressed with the K distribution to derive statistical parameters $\left(\tilde{v},\tilde{\mu }\right)$, or regressed with the Weibull distribution to derive statistical parameters $\left(\tilde{b},\tilde{c}\right)$. The outlier data are regressed with power-law distributions to derive power-law indices $\left({\tilde{b}}_{\ell 0},{\tilde{b}}_{\ell 1}\right)$. The details of these parts are presented in Section 2, Section 3 and Section 4.

Note that the third part of the proposed framework can be applied to the PDF derived from measured NRCS data, $\sigma_{0}^{\mathrm{obs}}$.

Due to the complicated ocean environment, rigorous formulation of normalized radar cross-sections (NRCSs), as in the canonical scattering problems, is impossible and impractical. Empirical models of NRCSs have been developed on vast measurement data and extensively used in practice. In this work, we take an intermediate approach by applying a physical-optics method to compute the NRCSs from individual realizations of sea-surface profiles generated with an empirical ocean-wave spectrum under specific wind conditions, then constitute a distribution of NRCS over multitudes of realizations, characterized with a few statistical parameters.

After reviewing a few state-of-the-art spectra, shown in Appendix A, the JONSWAP and Hwang spectra were picked to generate sea-surface realizations at low and high wind speeds, respectively. Other spectra can also be used in the proposed framework to generate sea-surface realizations suited to the scenarios of interest. Sea-surface realizations will become more realistic as more robust spectra become available.

To begin with, realizations of sea-surface profiles under a specific wind speed and direction are generated by applying a Monte Carlo method on either the JONSWAP or Hwang ocean-wave spectrum. Then, the backscattered field from a sea-surface realization under specific radar parameters is computed with the physical-optics method and transformed to a normalized radar cross-section (NRCS). The NRCS data over an ensemble of sea-surface realizations are compiled to form a probability density function (PDF), which is regressed with the K and Weibull distributions by applying a particle swarm optimization (PSO) method. The outlier regions of the PDF are fit with power-law distributions by applying a weighted linear regression method. The statistical parameters and the power-law indices are, thus, indirectly related to the designated wind conditions and the radar parameters.

A sea-surface profile is realized as follows. The sea-surface profile $h(\bar{r},t)$ is a function of time *t* and horizontal position $\bar{r}=\hat{x}x+\hat{y}y$, with $\hat{x}$ and $\hat{y}$ pointing in the east and north directions, respectively. The sea-surface profile can be represented as a generalized Fourier transform (GFT) of the amplitude spectrum $H({\bar{k}}_{w})$ as [43]

(1) h(r¯,t)=Re1(2π)2∫∫H(k¯w)e−jk¯w·r¯+jωwtdk¯w

with

(2) $$\begin{array}{ccc} & & \;H\left({\bar{k}}_{w}\right)=\;\int \int \;h\left(\bar{r},0\right)e^{j{\bar{k}}_{w}\cdot \bar{r}}d\bar{r} \end{array}$$

where ${\bar{k}}_{w}=\hat{x}k_{wx}+\hat{y}k_{wy}$ is the wavenumber vector of a plane-wave constituent, propagating with angular frequency $\omega_{w}$, which satisfies the dispersion relation of $\omega_{w}=\sqrt{gk_{w}}$ [43].

A two-dimensional wavenumber-directional energy spectrum is given by [44]

(3) $$\begin{array}{ccc} & & \;Q\left(k_{w},\phi \right)=k_{w}R\left({\bar{k}}_{w}\right)=\Theta \left(\phi \right)\Phi \left(k_{w}\right) \end{array}$$

where $R\left({\bar{k}}_{w}\right)={\left|H\left({\bar{k}}_{w}\right)\right|}^{2}/A$ is the power spectrum, *A* is the illuminated area, $\Phi (k_{w})$ is the one-dimensional wavenumber spectrum of an ocean wave (sea-surface profile), Θ(ϕ) is the azimuthal pattern which depends on the wind direction [45], $k_{w}=\left|{\bar{k}}_{w}\right|=\sqrt{k_{wx}^{2}+k_{wy}^{2}}$, and $\phi =\arctan(k_{wy}/k_{wx})$.

The Monte Carlo method is applied to generate a realization of a sea-surface profile based on discretizing (1), as

(4) hr([nx,ny],t)=ReΔkwxΔkwy(2π)2∑my=−Ny/2Ny/2−1∑mx=−Nx/2Nx/2−1H[mx,my]e−jmxΔkwxxbe−j2πmxnx/Nxe−jmyΔkwyybe−j2πmyny/Nyejωw[mx,my]tejϕ[mx,my]

with

(5) $$\begin{array}{ccc} & & \;x=x_{b}+n_{x}\Delta x,\;\;\;0\leq n_{x}\leq N_{x}-1\\ & & \;y=y_{b}+n_{y}\Delta y,\;\;\;0\leq n_{y}\leq N_{y}-1\\ & & \;k_{wx}=m_{x}\Delta k_{wx},\;\;\;-N_{x}/2\leq m_{x}\leq N_{x}/2-1\\ & & \;k_{wy}=m_{y}\Delta k_{wy},\;\;\;-N_{y}/2\leq m_{y}\leq N_{y}/2-1 \end{array}$$

$\phi \left[m_{x},m_{y}\right]\in \left[-\phi_{r},\phi_{r}\right]$ is a Gaussian random phase, with

(6) $$\begin{array}{ccc} & & \;\phi_{r}=\alpha_{c}\sqrt{|k_{wx}-k_{xp}{|}^{2}+{\left|k_{wy}-k_{yp}\right|}^{2}} \end{array}$$

where $\alpha_{c}$ is an empirical coefficient, and the maximum of $\left|H({\bar{k}}_{w})\right|$ appears at ${\bar{k}}_{w}=\left(k_{xp},k_{yp}\right)$.

Equation (1) shows the relation between the space–time sea-surface profile $h(\bar{r},t)$ and its amplitude spectrum $H({\bar{k}}_{w})$, which is the spectral component propagating with wavenumber vector ${\bar{k}}_{w}$ and temporal frequency $\omega_{w}$, satisfying the dispersion relation of $\omega_{w}=\sqrt{g|{\bar{k}}_{w}|}$ [43]. The amplitude spectrum $H({\bar{k}}_{w})$ is related to a snapshot of $h(\bar{r},t)$ at t=0 without loss of generality, by (2). The fact that $h(\bar{r},t)$ is real-valued implies that $H\left(-{\bar{k}}_{w}\right)=H^{*}\left({\bar{k}}_{w}\right)$, or $H({\bar{k}}_{w})$ is diagonally symmetric [7], if the Re{} operator is not imposed in (1).

The time evolution of $h(\bar{r},t)$ manifests water-wave features that move along the wind-blowing direction, which is achieved by multiplying the omnidirectional JONSWAP or Hwang spectrum with an azimuthal (angular spreading) pattern, like a cosine azimuthal pattern suggested in [46] and applied in [47]. In [44], a wave prediction model was developed by imposing a cosine azimuthal pattern on the JONSWAP spectrum under the constraint of $\int_{-\pi }^{\pi }\Theta \left(\phi \right)d\phi =1$. Either the two-dimensional JONSWAP or Hwang spectrum is substituted into (4) to generate realizations of sea-surface profiles, representing the interface between sea water and the atmosphere.

However, the two-dimensional spectrum embedding an azimuthal pattern no longer satisfies the condition of $H\left(-{\bar{k}}_{w}\right)=H^{*}\left({\bar{k}}_{w}\right)$. Thus, the conventional two-dimensional Fourier transform in (1) without the Re{} operator will give rise to complex-valued $h(\bar{r},t)$, and the generalized Fourier transform with the Re{} operator will generate real-valued $h(\bar{r},t)$ to comply with the observations.

In this work, the JONSWAP spectrum [48] is adopted to simulate sea-surface profiles under wind speeds of $U_{10}=6$–12 m/s [49], and the Hwang spectrum is adopted to simulate sea-surface profiles under wind speeds of $U_{10}\geq 10$ m/s [9].

### 2.1. JONSWAP Spectrum

The one-dimensional JONSWAP spectrum is given by [48]

(7) $$\begin{array}{ccc} & & \;\Phi_{J}\left(\omega_{w}\right)=\frac{\alpha g^{2}}{\omega_{w}^{5}}\left(2\pi \right)e^{-1.25{\left(\omega_{\max}/\omega_{w}\right)}^{4}}\\ & & \gamma^{\exp\{-{\left(\omega_{w}-\omega_{\max}\right)}^{2}/\left(2\sigma^{2}\omega_{\max}^{2}\right)\}} \end{array}$$

where α is an empirical parameter which is related to the fetch length *F* as [48]

(8) $$\begin{array}{ccc} & & \;\alpha =0.076{\left(\frac{Fg}{U_{10}^{2}}\right)}^{-0.22} \end{array}$$

and γ is the base of peak enhancement factor, *g* is the gravitational acceleration, $\omega_{\max}=g/U_{10}$ is the frequency of the spectral peak [6,44], $\sigma =\sigma_{a}$ if $\omega_{w}\leq \omega_{\max}$ and $\sigma =\sigma_{b}$ if $\omega_{w}>\omega_{\max}$, with $\sigma_{a}$ and $\sigma_{b}$ the left-side and right-side spectral widths, respectively, of the spectral peak. The JONSWAP spectrum can be transformed to the wavenumber domain as

(9) $$\begin{array}{ccc} \; & \; & \;\Phi_{J}\left(k_{w}\right)=\frac{\alpha \pi }{k_{w}^{3}}e^{-1.25{\left(\omega_{\max}/\omega_{w}\right)}^{4}}\gamma^{\exp\{-{\left(\omega_{w}-\omega_{\max}\right)}^{2}/\left(2\sigma^{2}\omega_{\max}^{2}\right)\}} \end{array}$$

**Figure 2:**
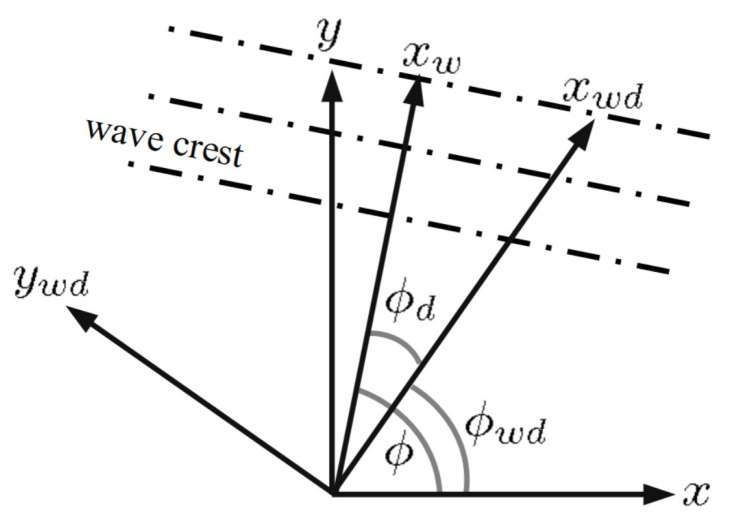
Relation between wind direction ($x_{wd}$) and sea-surface wave direction ($x_{w}$) [50]; *x* and *y* axes point east and north, respectively.

Figure 2 shows the relation between wind direction and sea-surface wave direction [50]. The *x* and *y* axes point in the east and north directions, respectively. The wind blows along the $x_{wd}$-axis, with an angle $\phi_{wd}$ from the *x*-axis. The sea-surface profile propagates along the $x_{w}$-axis, with an angle ϕ from the *x*-axis, and $\phi_{d}=\phi -\phi_{wd}$ is the angle between the moving direction ϕ of the sea-surface profile of interest and the wind-blowing direction $\phi_{wd}$. The azimuthal pattern Θ(ϕ) in the JONSWAP spectrum is given by [47]

(10) $$\begin{array}{ccc} & & \;\Theta_{J}\left(\phi \right)=\left\{\begin{array}{cc} 2\cos^{2}\left(\phi -\phi_{wd}\right),\;\;\; & |\phi -\phi_{wd}|\leq \pi /2\\ 0, & \mathrm{otherwise} \end{array}\right. \end{array}$$

which is contingent upon $\phi_{d}$.

**Figure 3:**
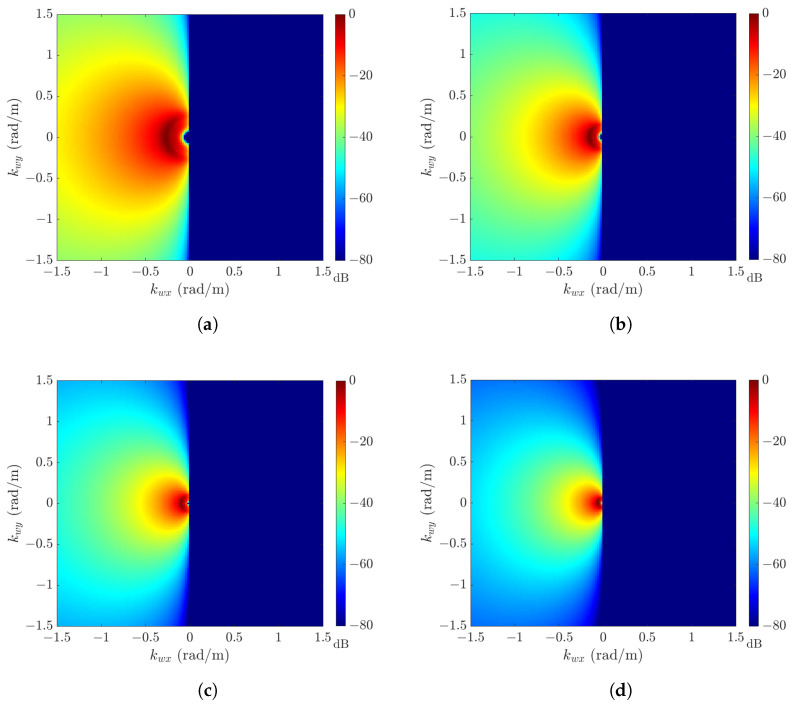
JONSWAP amplitude spectrum $H_{J}\left({\bar{k}}_{w}\right)$ (in dB), with default parameters in Table 1: (**a**) $U_{10}=6$ m/s; (**b**) $U_{10}=8$ m/s; (**c**) $U_{10}=10$ m/s; (**d**) $U_{10}=12$ m/s.

Table 1 lists the default parameters used to generate sea-surface profiles with the JONSWAP spectrum. Figure 3 shows the JONSWAP amplitude spectrum $H_{J}\left({\bar{k}}_{w}\right)$, with the default parameters listed in Table 1 and wind speeds of $U_{10}=6,8,10$, and 12 m/s. Each amplitude spectrum is normalized against its maximum amplitude, which is listed in Table 2. As the wind speed increases, the maximum amplitude increases and the dominant spectral region shrinks.

**Figure 4:**
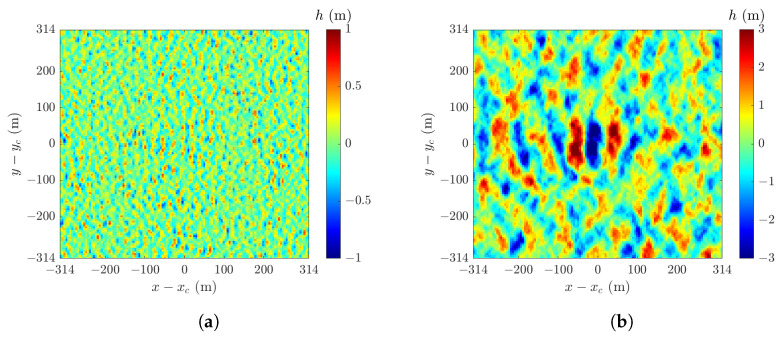
Sample snapshots of sea-surface realization $h_{Jr}\left(\bar{r},t\right)$ with JONSWAP spectrum: (**a**) $U_{10}=6$ m/s; (**b**) $U_{10}=12$ m/s.

Figure 4 shows sample snapshots of sea-surface realizations $h(\bar{r},t)$ computed with (4) in an area of 628 m × 628 m, under $U_{10}=6$ m/s and 12 m/s. Each realization manifests water-wave features that move along the wind-blowing direction. As $U_{10}$ is increased from 6 m/s to 12 m/s, the crest-to-trough amplitude increases, the wave-fronts perpendicular to the wind direction become more conspicuous.

**Figure 5:**
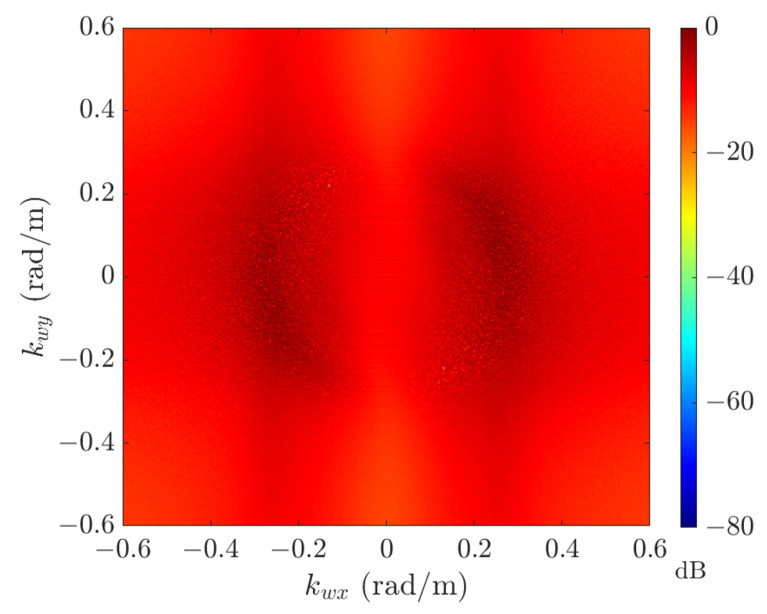
Amplitude spectrum reconstructed from realizations of sea-surface profile generated with JONSWAP amplitude spectrum in Figure 3a; $U_{10}=6$ m/s.

To confirm that the realizations follow the specified two-dimensional spectrum, we generate multiple snapshots from multiple realizations, compute the autocorrelation of each snapshot, and take the two-dimensional Fourier transform with respect to the spatial offset to derive a sample spectrum. Figure 5 shows the ensemble average over multiple sample spectra, under $U_{10}=6$ m/s, expecting to reconstruct the amplitude spectrum shown in Figure 3a. The reconstructed spectrum reveals not only the original amplitude spectrum, but also its diagonally symmetric image, because each sample spectrum is derived from a snapshot of $h(\bar{r},t)$ at a fixed time instant, which is real-valued and implies a diagonally symmetric sample spectrum. Note that a time-frozen snapshot does not manifest movement of wave features. The Re{} operator in the generalized Fourier transform of (1) implies a real-valued $h(\bar{r},t)$ while preserving the movement of wave features.

The significant wave height (SWH) used to characterize sea-surface profiles under the specific $U_{10}$ is also compared with its empirical counterpart specified in the Douglas (DG) sea-state table [52]. The significant wave height is defined as $h_{s}=4\sigma_{h}$ [53], with $\sigma_{h}=\sqrt{\langle h_{J}^{2}\rangle }$ the root-mean-square of the sea-surface profiles, namely,

(11) $$\begin{array}{ccc} & & \;\sigma_{h}={\left\{\frac{1}{N_{a}}\sum_{n=1}^{N_{a}}\frac{1}{A}\;\int \int \;h_{J}^{2}\left(\bar{r},t_{n}\right)d\bar{r}\right\}}^{1/2} \end{array}$$

where $N_{a}$ is the number of snapshots, and the integral over $\bar{r}$ can be implemented as a sum over $N_{x}\times N_{y}$ spatial cells in the illuminated area *A*.

Table 3 lists the SWHs at $U_{10}=6,8,10$, and 12 m/s, which fall in the range of SWH specified in the DG sea-state table [52].

### 2.2. Hwang Spectrum

The one-dimensional Hwang spectrum is given by [54,55,56]

(12) $$\begin{array}{ccc} & & \;\Phi_{H}\left(k_{w}\right)=\frac{1}{k_{w}^{3}}B_{H}\left(k_{w}\right) \end{array}$$

with

(13) $$\begin{array}{ccc} & & \;B_{H}\left(k_{w}\right)=A_{H}\left(k_{w}\right){\left[\frac{u_{*}}{V_{w}\left(k_{w}\right)}\right]}^{a_{H}\left(k_{w}\right)} \end{array}$$

where $u_{*}$ is the air friction velocity, and $V_{w}$ is the phase velocity of sea-surface profile (ocean wave). The amplitude $A_{H}\left(k_{w}\right)$ and the exponent $a_{H}\left(k_{w}\right)$ are given by fifth-order polynomials of $k_{\ln}=\ln k_{w}$ as [54]

(14) $$\begin{array}{ccc} & & \;A_{H}\left(k_{w}\right)=-3.862\times 10^{-5}k_{\ln}^{5}+7.991\times 10^{-4}k_{\ln}^{4}\\ & & -6.417\times 10^{-3}k_{\ln}^{3}+2.342\times 10^{-2}k_{\ln}^{2}\\ & & -3.668\times 10^{-2}k_{\ln}+2.898\times 10^{-2}\\ & & \;a_{H}\left(k_{w}\right)=-5.213\times 10^{-4}k_{\ln}^{5}+1.524\times 10^{-2}k_{\ln}^{4}\\ & & -1.358\times 10^{-1}k_{\ln}^{3}+5.865\times 10^{-1}k_{\ln}^{2}\\ & & -1.167k_{\ln}+1.136 \end{array}$$

with $k_{w\ell }\leq k_{w}\leq k_{wh}$, where $k_{w\ell }=1.5$ rad/m and $k_{wh}=100$ rad/m.

The expressions of $A_{H}\left(k_{w}\right)$ and $a_{H}\left(k_{w}\right)$ in the wavenumber ranges of $0\leq k_{w}<k_{w\ell }$ and $k_{wh}<k_{w}<\infty$ can be found in [54], hence are not listed here.

The azimuthal pattern $\Theta_{J}\left(\phi \right)$ in (10) is adopted if $10\leq U_{10}<14$ m/s. To make the significant wave height (SWH) derived from the realizations of sea-surface profile consistent with those suggested in the Douglas sea-state table, under $14\leq U_{10}\leq 20$ m/s, we adopt the azimuthal pattern [57]

(15) $$\begin{array}{ccc} & & \;\Theta_{H}\left(k_{w},\phi \right)=\beta_{1}\left(k_{w}\right)+\Delta_{1}\left(k_{w}\right)\cos\left(\phi -\phi_{wd}\right)\\ & & +\mathrm{sgn}\left(\pi /2-\phi_{d}\right)\Delta_{2}\left(k_{w}\right)\cos\left[2\left(\phi -\phi_{wd}\right)\right] \end{array}$$

with

(16) $$\begin{array}{ccc} & & \;\beta_{1}\left(k_{w}\right)=\left\{\begin{array}{cc} 1+2\Delta_{2}\left(k_{w}\right),\; & |\phi -\phi_{wd}|\leq \pi /2\\ 1, & |\phi -\phi_{wd}|>\pi /2 \end{array}\right. \end{array}$$

where sgn(x)=±1 if x≷0,

(17) $$\begin{array}{ccc} & & \;\Delta_{1}\left(k_{w}\right)=\exp\left\{-4{\left[\log\left(\frac{u_{*}}{1.5V_{w}}\right)\right]}^{2}\right\}(-4.66\times 10^{-7}k_{w}^{2}\\ & & +4.55\times 10^{-4}k_{w}+6.78\times 10^{-2}) \end{array}$$

is the upwind–downwind ratio [58],

(18) $$\begin{array}{c} \Delta_{2}\left(k_{w}\right)=\tanh\left\{a_{0}+a_{p}{\left(V_{w}/V_{p}\right)}^{2.5}+a_{\min}{\left(V_{\min}/V_{w}\right)}^{2.5}\right\} \end{array}$$

is the upwind–crosswind ratio [51]; $a_{0}=\ln2/4$, $a_{p}=4$, $a_{\min}=0.13u_{*}/V_{\min}$, $V_{\min}=0.23$ m/s, and  $V_{p}=V_{w}\left(k_{p}\right)$ (m/s) is the phase speed at the spectral peak [51]

(19) $$\begin{array}{ccc} & & \;k_{p}=\frac{g}{U_{10}^{2}}\Omega_{c}^{2} \end{array}$$

where $\Omega_{c}$ is the inverse wave-age parameter.

**Figure 6:**
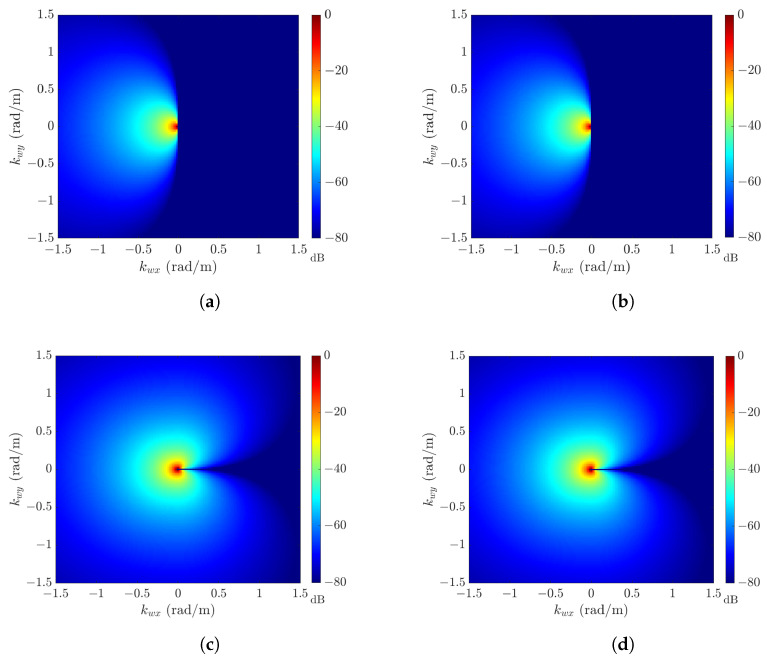
Hwang amplitude spectrum $H_{H}\left({\bar{k}}_{w}\right)$ (in dB), with default parameters in Table 1: (**a**) $U_{10}$ = 10 m/s; (**b**) $U_{10}$ = 12 m/s; (**c**) $U_{10}$ = 16 m/s; (**d**) $U_{10}$ = 20 m/s.

Figure 6 shows the amplitude spectra $H_{H}\left({\bar{k}}_{w}\right)$ at wind speeds of $U_{10}=10,12,16$, and 20 m/s, with the default parameters listed in Table 1. Each amplitude spectrum is normalized against its maximum value, which is listed in Table 4. The amplitude spectra at $U_{10}=10$ and 12 m/s manifest patterns similar to $\Theta_{J}\left(\phi \right)$ in (10), and those at $U_{10}=16$ and 20 m/s manifest patterns similar to $\Theta_{H}\left(\phi \right)$ in (15).

**Figure 7:**
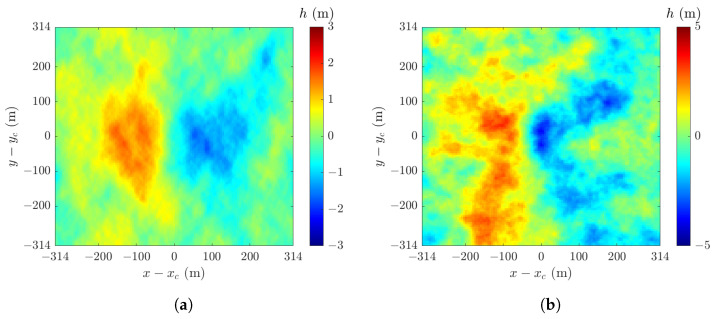
Samples of sea-surface realization $h_{Hr}\left(\bar{r},t\right)$ with Hwang spectrum: (**a**) $U_{10}=12$ m/s; (**b**) $U_{10}=20$ m/s.

Figure 7 shows samples of sea-surface realization in an area of 628 m × 628 m, with $U_{10}=12$ m/s and 20 m/s. By comparing with Figure 4, the features of long waves become more conspicuous, and the size of wave fronts and crest-to-trough amplitudes increase with the wind speed.

Table 5 lists the SWHs at $U_{10}=10,12,16$, and 20 m/s. It is observed that the SWH at $U_{10}=10$ m/s falls within the suggested range in the DG sea-state table, while those at $U_{10}=12,16$, and 20 m/s are close to the lower end of their suggested ranges in the DG sea-state table [52].

## 3. Computation of Normalized Radar Cross-Section

**Figure 8:**
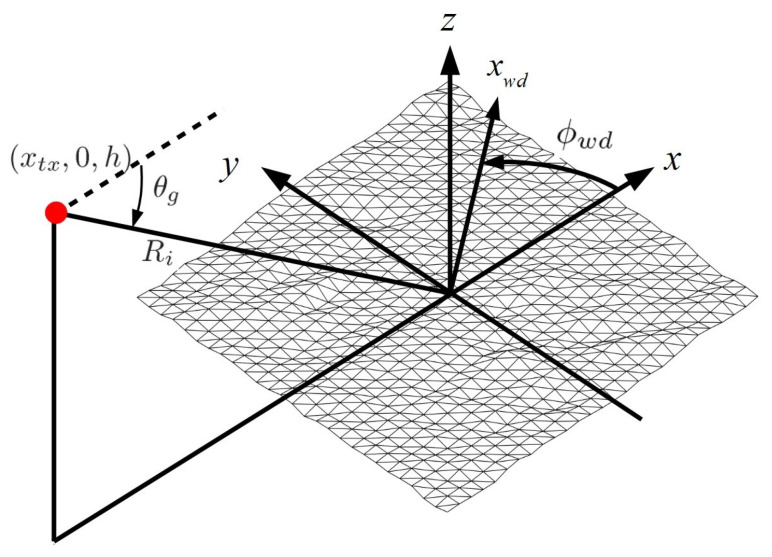
Schematic of computing radar backscattered field from sea-surface profile.

Figure 8 shows the schematic of computing the radar backscattered field from a sea-surface profile, which is modeled with triangular patches. The radar is located at $\left(x_{tx},0,h\right)$, the slant range between the radar and the center of the target area is $R_{i}$, and the angle between the downwind direction $x_{wd}$ and the *x*-axis is $\phi_{wd}$. A continuous electromagnetic wave is radiated towards the target area, at a grazing angle of $\theta_{g}$. The backscattered electric fields from all the triangular patches are computed by using the physical-optics (PO) method, and then, transformed to the NRCS from the target region.

**Figure 9:**
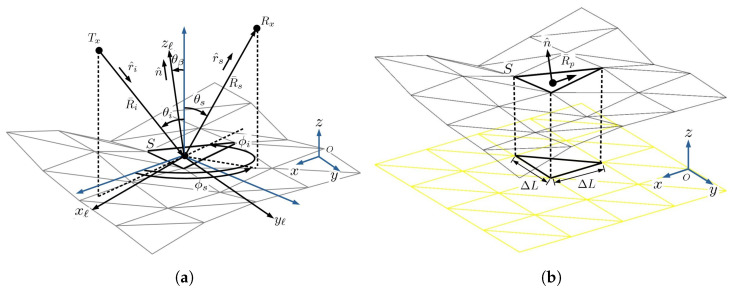
(**a**) Schematic of a plane wave incident upon a sea-surface profile modeled with triangular patches. (**b**) Projection of triangle *S* onto xy-plane.

Figure 9a shows the schematic of a plane wave incident upon the sea-surface profile modeled with triangular patches. Figure 9b shows that the sea-surface profile is projected onto a grid of triangular patches on the xy-plane, each with edge length of ΔL. The centroid of a triangle *S* is chosen as the origin of a local Cartesian coordinate system $\left(x_{\ell },y_{\ell },z_{\ell }\right)$, with a normal vector of $\hat{n}={\hat{z}}_{\ell }$. The incident and the scattering directions are specified in the global (x,y,z) coordinate by $\left(\theta_{i},\phi_{i}\right)$ and $\left(\theta_{s},\phi_{s}\right)$, respectively. The unit vectors in the scattering direction and the incident direction are ${\hat{r}}_{s}$ and ${\hat{r}}_{i}$, respectively, ${\bar{R}}_{i}$ is the range vector from the transmitter $T_{x}$ to the centroid of *S*, and ${\bar{R}}_{s}$ is the range vector from the latter to the receiver $R_{x}$.

The angle between the *z*-axis and the local normal vector $\hat{n}$ is $\theta_{\beta }$. The local incident angle with respect to $\hat{n}$ is $\theta_{n}=\theta_{i}-\theta_{\beta }$. The scattering field from *S* is computed as an integral over its projection in the xy-plane as [59]

(20) $$\begin{array}{ccc} & & \;E_{pq}^{s}\left({\bar{R}}_{s}\right)=jk\frac{e^{-jk(|{\bar{R}}_{i}|+|{\bar{R}}_{s}|)}}{4\pi |{\bar{R}}_{i}\left|\right|{\bar{R}}_{s}|}E_{0}U_{pq}\int_{S}e^{jk\left({\hat{r}}_{s}-{\hat{r}}_{i}\right)\cdot {\bar{R}}_{p}}ds \end{array}$$

where *k* is the wavenumber of the radar wave, $E_{0}$ is the magnitude of the incident electric field, ${\bar{R}}_{p}={\hat{x}}_{\ell }x_{\ell }+{\hat{y}}_{\ell }y_{\ell }$, and $U_{pq}$ is the polarization factor, given by [59]

(21) $$\begin{array}{ccc} & & \;U_{pq}=\frac{1}{\sqrt{\zeta_{x}^{2}+\zeta_{y}^{2}+1}}[a_{pq0}+a_{pq1}\left(\zeta_{x}\cos\phi_{i}+\zeta_{y}\sin\phi_{i}\right)\\ & & +a_{pq2}{\left(\zeta_{x}\cos\phi_{i}+\zeta_{y}\sin\phi_{i}\right)}^{2}],\;\;p,q=v,h \end{array}$$

where ζ(x,y) is the sea-surface profile, $\zeta_{x}=\partial \zeta \left(x,y\right)/\partial x$ and $\zeta_{y}=\partial \zeta \left(x,y\right)/\partial y$ are the slopes of ζ(x,y) in the *x* and *y* directions, respectively. The coefficients $a_{pqn}$, with p,q=v,h and n=0,1,2, are derived in terms of $\left(\theta_{i},\phi_{i}\right)$, $\left(\theta_{s},\phi_{s}\right)$, and the Fresnel reflection coefficients are approximated as [59]

(22) $$\begin{array}{ccc} & & \;\Gamma_{h}≃\Gamma_{h0}+\Gamma_{h1}\left(\zeta_{x}\cos\phi_{i}+\zeta_{y}\sin\phi_{i}\right) \end{array}$$

(23) $$\begin{array}{ccc} & & \;\Gamma_{v}≃\Gamma_{v0}+\Gamma_{v1}\left(\zeta_{x}\cos\phi_{i}+\zeta_{y}\sin\phi_{i}\right) \end{array}$$

The incident electric field ${\bar{E}}^{i}$ from the transmitter $T_{x}$ is given by

(24) $$\begin{array}{ccc} & & \;{\bar{E}}^{i}={\hat{e}}_{i}E_{0}\frac{e^{-jk|{\bar{R}}_{i}-{\bar{R}}_{p}|}}{|{\bar{R}}_{i}-{\bar{R}}_{p}|}≃{\hat{e}}_{i}E_{0}\frac{e^{-jk|{\bar{R}}_{i}|}e^{-jk{\hat{r}}_{i}\cdot {\bar{R}}_{p}}}{|{\bar{R}}_{i}|} \end{array}$$

where ${\hat{e}}_{i}$ is the unit polarization vector of the incident field. In (20), the electric field scattered from the *n*th triangular patch is reduced to

(25) $$\begin{array}{ccc} & & \;E_{pqn}^{s}\left({\bar{R}}_{sn}\right)=a_{n}\left({\bar{R}}_{sn}\right)e^{j\phi_{n}\left({\bar{R}}_{sn}\right)} \end{array}$$

where

(26) $$\begin{array}{ccc} & & \;a_{n}\left({\bar{R}}_{sn}\right)=\frac{|{\bar{E}}^{i}|}{|\bar{R}|}b_{n}\left({\bar{R}}_{sn}\right)\\ & & \;b_{n}\left({\bar{R}}_{sn}\right)=\frac{jkU_{pqn}}{4\pi }\int_{S}e^{jk\left({\hat{r}}_{sn}-{\hat{r}}_{in}\right)\cdot {\bar{R}}_{p}}ds\\ & & \;\phi_{n}\left({\bar{R}}_{sn}\right)=-k(|{\bar{R}}_{in}\left|+\right|{\bar{R}}_{sn}\left|\right) \end{array}$$

By making further approximations that $|{\bar{R}}_{in}\left|=\right|{\bar{R}}_{sn}\left|≃\right|\bar{R}|$ and $|{\bar{E}}_{n}^{i}\left|≃\right|{\bar{E}}^{i}|$, the total scattered electric field in (20) is computed as the sum of contributions from all the triangular patches in the target area as [60]

(27) $$\begin{array}{ccc} & & \;E_{pq}^{s}\left({\bar{R}}_{s}\right)≃\frac{|{\bar{E}}^{i}|}{|\bar{R}|}\sum_{n=1}^{N}b_{n}\left({\bar{R}}_{sn}\right)e^{j\phi_{n}\left({\bar{R}}_{sn}\right)} \end{array}$$

Then, the radar cross-section (RCS) is computed as

(28) $$\begin{array}{ccc} & & \;\sigma =4\pi |\bar{R}{|}^{2}\frac{|E_{pq}^{s}\left({\bar{R}}_{s}\right){|}^{2}}{|{\bar{E}}^{i}{|}^{2}}\;\left(m^{2}\right)\\ & & \;=4\pi {\left|\sum_{n=1}^{N}b_{n}\left({\bar{R}}_{sn}\right)e^{j\phi_{n}\left({\bar{R}}_{sn}\right)}\right|}^{2}\;\left(m^{2}\right) \end{array}$$

The normalized RCS (NRCS) is the RCS per unit scattering area [61], namely,

(29) $$\begin{array}{ccc} & & \;\sigma_{0}=\frac{\sigma }{A} \end{array}$$

where $A=L^{2}$ is the size of the target area, which is a square of edge length *L*.

The dielectric constant of sea water is determined by using the double-Debye dielectric model (D3M) as [61]

(30) $$\begin{array}{ccc} & & \;\epsilon_{rw}=\epsilon_{w}^{\prime }-j\epsilon_{w}^{\prime \prime } \end{array}$$

with

(31) $$\epsilon_{w}^{\prime }=\epsilon_{w\infty }+\frac{\epsilon_{w0}-\epsilon_{w1}}{1+{\left(2\pi f\tau_{w1}\right)}^{2}}+\frac{\epsilon_{w0}-\epsilon_{w\infty }}{1+{\left(2\pi f\tau_{w2}\right)}^{2}}$$

(32) $$\begin{array}{c} \epsilon_{w}^{\prime \prime }=\frac{2\pi f\tau_{w1}\left(\epsilon_{w0}-\epsilon_{w1}\right)}{1+{\left(2\pi f\tau_{w1}\right)}^{2}}+\frac{2\pi f\tau_{w2}\left(\epsilon_{w0}-\epsilon_{w\infty }\right)}{1+{\left(2\pi f\tau_{w2}\right)}^{2}}\\ \;+\frac{\sigma_{i}}{2\pi \epsilon_{0}f} \end{array}$$

where *f* is the radiation frequency, $\epsilon_{0}$ is the permittivity of free space, and the empirical formulas of the other parameters can be found in [61].

Figure 4 and Figure 7 show a few samples of sea-surface profiles generated with the JONSWAP and the Hwang spectra, respectively. The NRCSs of over 40,000 realizations are computed to form a convergent PDF of NRCS data. Empirically, the background of sea clutter can be reasonably well modeled in terms of tilt-modulated Bragg scattering, whereas the spikes can be modeled via the scattering from steepened and/or breaking waves. These features account for some salient signatures in the sea clutter.

In this work, an alternative approach is proposed by generating sea-surface profiles in terms of an empirical ocean-wave spectrum. This alternative is consistent with the empirical model just mentioned as long as the sea-surface features (tilt-modulated surface, steepened and/or breaking waves) are manifested in the sea-surface realizations. The samples in Figure 4 manifest geometrical features that may cause Bragg scattering via steepened and/or breaking waves. The same argument applies to surface gravity waves (swells) and capillary waves (ripples). Different geometrical features may be highlighted by adopting different ocean-wave spectra.

In this work, each realization of a sea-surface profile is approximated with a set of triangular patches, as shown in Figure 8 and Figure 9, before applying the physical-optics (PO) method to compute the NRCS. The PO method demands that a triangular patch cannot be too small compared with the wavelength. Thus, the swells can be well represented, but maybe not the ripples. A more delicate electromagnetic model is preferred to better account for the ripples, which may take higher computational cost.

## 4. Estimation of Statistical Parameters on PDF of NRCS

The NRCSs computed on an ensemble of sea-surface realizations are compiled to form a probability density function (PDF), which is then regressed with the Weibull and K distributions [17]. However, the outlier regions with very small and very large NRCSs, respectively, cannot regress well with the K or Weibull distributions. The Pareto distribution [30] and other compound models like KK [62], WW [62], and K+Rayleigh [40] have been adopted to characterize the non-Bragg scattering caused by sea spikes.

However, a given PDF may lead to multiple sets of statistical parameters in a compound model, which implies that a slight change in the distribution of NRCSs may overly perturb the statistical parameters, hampering the prediction of the latter under different operational conditions.

In this work, the K and Weibull distributions are adopted to regress the PDF of the simulated NRCS data, and the power-law is used to regress the outlier regions of very large and very small NRCSs.

The PDF of an NRCS, $\sigma_{0}^{\prime }=10\log_{10}\sigma_{0}$ (in dB), is approximated from the set of simulated NRCS data as [63]

(33) $$\begin{array}{ccc} & & \;p^{\prime }\left(\sigma_{0}^{\prime }\right)=\frac{H(\sigma_{0}^{\prime })}{\Delta \sigma_{0}^{\prime }N_{\mathrm{rd}}} \end{array}$$

where $N_{\mathrm{rd}}$ is the total number of NRCS data, $\Delta \sigma_{0}^{\prime }$ is the bin width in dB scale, and $H(\sigma_{0}^{\prime })$ is the number of NRCS data falling in $\left[\sigma_{0}^{\prime }-\Delta \sigma_{0}^{\prime }/2,\sigma_{0}^{\prime }+\Delta \sigma_{0}^{\prime }/2\right]$. The ratio $H\left(\sigma_{0}^{\prime }\right)/N_{\mathrm{rd}}$ approximates the probability of $\sigma_{0}^{\prime }\in \left[\sigma_{0}^{\prime }-\Delta \sigma_{0}^{\prime }/2,\sigma_{0}^{\prime }+\Delta \sigma_{0}^{\prime }/2\right]$, which is equal to the PDF $p^{\prime }\left(\sigma_{0}^{\prime }\right)$ multiplied by the bin width $\Delta \sigma_{0}^{\prime }$.

The PDF of the K distribution is given by [64]

(34) $$\begin{array}{ccc} & & \;p_{K}\left(\sigma_{0};v,\mu \right)=\int_{0}^{\infty }p_{\mathrm{cr}}\left(\sigma_{0}|\xi \right)p_{\chi }\left(\xi \right)d\xi =\frac{2}{\sigma_{0}\Gamma \left(v\right)\Gamma \left(m\right)}\\ & & {\left(\frac{mv}{\mu }\sigma_{0}\right)}^{(v+m)/2}K_{v-m}\left(2\sqrt{\frac{mv}{\mu }\sigma_{0}}\right) \end{array}$$

where *v* and μ are the statistical parameters to be estimated by regression, *m* is the number of looks, and m=1 is adopted in this work, $K_{\nu }\left(x\right)$ is the νth-order modified Bessel function of the second kind [65],

(35) $$\begin{array}{ccc} & & \;p_{\chi }\left(\xi \right)={\left(\frac{v}{\mu }\right)}^{v}\frac{\xi^{v-1}}{\Gamma (v)}e^{-v\xi /\mu },\;\xi \geq 0 \end{array}$$

is the chi distribution, and

(36) $$\begin{array}{ccc} & & \;p_{\mathrm{cr}}\left(\sigma_{0}|\xi \right)={\left(\frac{m}{\xi }\right)}^{m}\frac{\sigma_{0}^{m-1}}{\Gamma (m)}e^{-m\sigma /\xi },\;\sigma_{0}\geq 0 \end{array}$$

is the conditional Rayleigh distribution. To regress with (33) in the dB scale, (34) is transformed to the dB scale as

(37) $$\begin{array}{ccc} & & \;p_{K}^{\prime }\left(\sigma_{0}^{\prime }\right)=p_{K}\left(\sigma_{0}\right)\sigma_{0}\frac{\ln10}{10} \end{array}$$

The PDF of the Weibull distribution is given by [66]

(38) $$\begin{array}{ccc} & & \;p_{W}\left(\sigma_{0}\right)=\frac{c\sigma_{0}^{c-1}}{b^{c}}e^{-{\left(\sigma_{0}/b\right)}^{c}} \end{array}$$

where *b* and *c* are are the statistical parameters to be estimated by regression. To regress with (33) in the dB scale, (38) is transformed to

(39) $$\begin{array}{ccc} & & \;p_{W}^{\prime }\left(\sigma_{0}^{\prime }\right)=p_{W}\left(\sigma_{0}\right)\sigma_{0}\frac{\ln10}{10} \end{array}$$

### 4.1. Validation with Measurement Data

**Figure 10:**
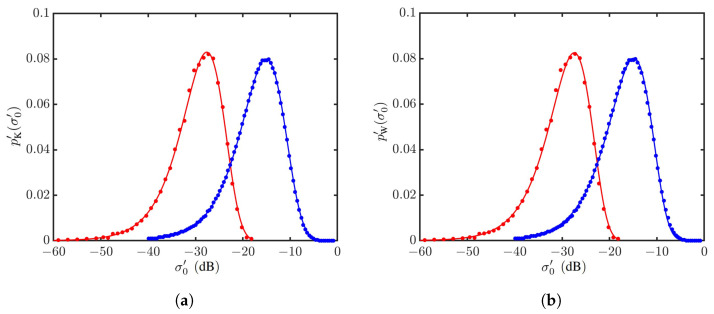
PDFs of NRCSs at vv polarization: $U_{10}=9.3$ m/s, $\theta_{g}=38.7^{\circ }$, $\phi_{wd}=68^{\circ }$. (**a**) •: Data of run34683 [62], **—**: regressed with K distribution; •: simulated NRCS data, **—**: regressed with K distribution. (**b**) •: Data of run34683 [62], **—**: regressed with Weibull distribution; •: simulated NRCS data, **—**: regressed with Weibull distribution.

The PDF of simulated NRCS data, $p^{\prime }\left(\sigma_{0}^{\prime }\right)$, is validated by comparing with its counterpart PDF of measured sea clutter in an Ingara dataset [62], as shown in Figure 10. The PDF $p^{\prime }\left(\sigma_{0}^{\prime }\right)$ matches well with its counterpart of measurement in shape and size, except for a lateral shift, which is attributed to unknown normalization and calibration in the measurement data.

### 4.2. Particle Swarm Optimization

**Figure 11:**
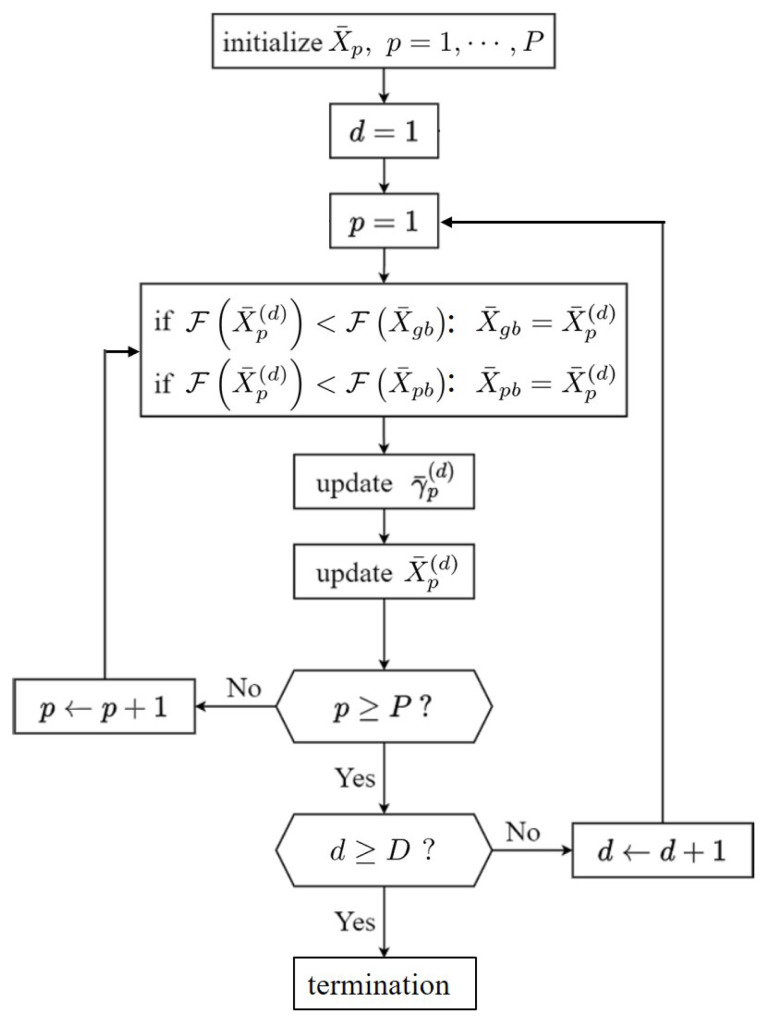
Flowchart for estimating the statistical parameters of a specific probability density function with particle swarm optimization (PSO) algorithm.

Figure 11 shows the flowchart for estimating the statistical parameters of (v,μ) in the K distribution or (b,c) in the Weibull distribution by applying a particle swarm optimization (PSO) algorithm to the PDF derived from the set of simulated NRCS data.

A total of *P* pseudo-particles are guided by the PSO algorithm to move around in a two-dimensional space, in which the position vector ${\bar{X}}_{p}$ of the *p*th particle is defined as

(40) $$\begin{array}{ccc} & & \;{\bar{X}}_{p}=\left\{\begin{array}{c} {}[v_{p},\mu_{p}],\;\;\;K\mathrm{distribution}\\ b_{p},c_{p}],\;\;\;\;\mathrm{Weibull}\mathrm{distribution} \end{array}\right. \end{array}$$

The initial values of $\left\{{\bar{X}}_{p}\right\}$ are randomly picked in the ranges of $0.1\leq v_{p}\leq 25$, $0\leq \mu_{p}\leq 2\mu$, $0\leq b_{p}\leq 10$, and $0\leq c_{p}\leq 10$.

Next, the fitness functions associated with the K and Weibull distributions are defined as

(41) $$\begin{array}{ccc} & & \;F_{K}\left({\bar{X}}_{p}\right)=\int \left|p_{K}^{\prime }\left(\sigma_{0}^{\prime },{\bar{X}}_{p}\right)-p^{\prime }\left(\sigma_{0}^{\prime }\right)\right|W\left(\sigma_{0}^{\prime }\right)d\sigma_{0}^{\prime } \end{array}$$

(42) $$\begin{array}{ccc} & & \;F_{W}\left({\bar{X}}_{p}\right)=\int \left|p_{W}^{\prime }\left(\sigma_{0}^{\prime },{\bar{X}}_{p}\right)-p^{\prime }\left(\sigma_{0}^{\prime }\right)\right|W\left(\sigma_{0}^{\prime }\right)d\sigma_{0}^{\prime } \end{array}$$

respectively, where $W\left(\sigma_{0}^{\prime }\right)=H\left(\sigma_{0}^{\prime }\right)/N_{\mathrm{rd}}$ is used as the weighting function.

The positions of all the particles are iterated by *D* rounds. In each iteration, the position of the *p*th particle reaching its lowest fitness function so far is registered as ${\bar{X}}_{pb}$, and the position with the lowest fitness function ever reached by all the *P* particles is registered as ${\bar{X}}_{gb}$. The velocity of the *p*th particle in iteration *d* is updated as

(43) $$\begin{array}{ccc} & & \;{\bar{\gamma }}_{p}^{\left(d\right)}=h_{w}{\bar{\gamma }}_{p}^{\left(d-1\right)}+c_{\ell }{\bar{\beta }}_{\ell }⊙\left({\bar{X}}_{pb}-{\bar{X}}_{p}^{\left(d\right)}\right)\\ & & +c_{g}{\bar{\beta }}_{g}⊙\left({\bar{X}}_{gb}-{\bar{X}}_{p}^{\left(d\right)}\right) \end{array}$$

where ${\bar{\beta }}_{\ell },{\bar{\beta }}_{g}\in R^{2}$ are two-dimensional vectors, with each component a random number of uniform distribution in [0,1]; $h_{w}=0.4$ is the inertia weight, $c_{\ell }=c_{g}=2$ are empirical constants, and ⊙ is the Hadamard product operator. The position of the *p*th particle is then updated as

(44) $$\begin{array}{ccc} & & \;{\bar{X}}_{p}^{\left(d+1\right)}={\bar{X}}_{p}^{\left(d\right)}+{\bar{\gamma }}_{p}^{\left(d\right)} \end{array}$$

The population size is set to P=20. The PSO algorithm halts when *d* reaches D=40 for the K distribution and D=60 for the Weibull distribution.

The PSO method has been widely used in many disciplines and is used as an auxiliary tool in this work. Other methods can be used to estimate these statistical parameters. It is worth mentioning that the weighting function $W\left(\sigma_{0}^{\prime }\right)=H\left(\sigma_{0}^{\prime }\right)/N_{\mathrm{rd}}$ adopted in (41) and (42) is the posteriori information, which is the probability of observed data falling in the designated $\sigma_{0}^{\prime }$ bin. This weighting function bears the same spirit as in the maximum-likelihood estimation, which turns out to improve the goodness of fit.

### 4.3. Power-Law Distribution

The power-law distribution has been observed in the outlier region of many physical quantities [67]. The PDFs of NRCSs with very large and very small values appear to follow the power-law distribution. The outlier regions are specified as $\left[d_{s},\infty \right)$ (dB) and $\left(-\infty ,d_{w}\right]$ (dB), respectively, with the thresholds $d_{s}$ and $d_{w}$ selected by observation.

Each outlier region is divided into $M_{b}$ bins, with the PDF value $y_{\ell m}$ (dB) in the *m*th bin linearly regressed as

(45) $$\begin{array}{ccc} & & \;y_{\ell m}=b_{\ell 0}+b_{\ell 1}\sigma_{0m}^{\prime } \end{array}$$

where $1\leq m\leq M_{b}$, and $\left(b_{\ell 0},b_{\ell 1}\right)$ are the regression coefficients, which are determined by minimizing the sum of squared errors as

(46) $$\begin{array}{ccc} & & \;\left({\tilde{b}}_{\ell 0},{\tilde{b}}_{\ell 1}\right)=\arg\min_{\left(b_{\ell 0},b_{\ell 1}\right)}\sum_{m=1}^{M_{b}}W_{\ell m}{\left(y_{\ell m}-p_{\mathrm{dB}m}^{\prime }\right)}^{2} \end{array}$$

where $p_{\mathrm{dB}}^{\prime }\left(\sigma_{0}^{\prime }\right)=10\log_{10}p^{\prime }\left(\sigma_{0}^{\prime }\right)$, and $W_{\ell m}$ is the value of weighting function

(47) $$\begin{array}{ccc} & & \;W_{\ell }\left(\sigma_{0}^{\prime }\right)=\left\{\begin{array}{c} H\left(\sigma_{0}^{\prime }\right)/N_{w},\;\;\;\sigma_{0}^{\prime }\leq d_{w}\;\left(\mathrm{dB}\right)\\ H\left(\sigma_{0}^{\prime }\right)/N_{s},\;\;\;\sigma_{0}^{\prime }\geq d_{s}\;\left(\mathrm{dB}\right) \end{array}\right. \end{array}$$

in the *m*th bin, $N_{w}$ and $N_{s}$ are the numbers of NRCS data with $\sigma_{0}^{\prime }\leq d_{w}$ and $\sigma_{0}^{\prime }\geq d_{s}$, respectively.

## 5. Simulations and Discussion

In this section, realizations of sea-surface profiles are generated under different wind speeds and wind directions, with either the JONSWAP or Hwang spectrum. The NRCS from each sea-surface realization is computed by using the default radar parameters listed in Table 6. The NRCS data from all the realizations are compiled to form a PDF, which is then regressed with the K and Weibull distributions to derive two statistical parameters of each distribution by using the particle swarm optimization method. The power-law indices in the two outlier regions are determined by applying the weighted linear regression method. Then, the effects of wind speed, wind direction, grazing angle, and polarization on these statistical parameters are investigated.

### 5.1. Effects of Wind Speed

**Figure 12:**
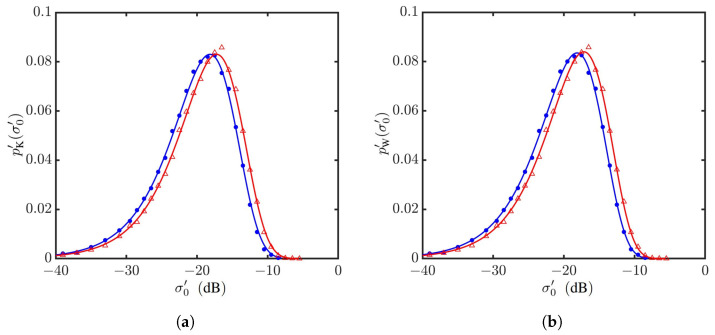
PDFs of NRCSs at vv polarization, with default parameters listed in Table 1 and Table 6. •: Generated with JONSWAP spectrum, $U_{10}=6$ m/s; Δ: generated with Hwang spectrum, $U_{10}=20$ m/s. **—**: Regressed on •; **—**: regressed on Δ. (**a**) Regressed with K distribution; (**b**) regressed with Weibull distribution.

The effects of wind speed on an NRCS are studied by computing $\sigma_{0vv}^{\prime }$’s over 40,000 realizations of sea-surface profiles at each wind speed of interest. Figure 12a shows the K distributions, regressed with the PDFs formed at $U_{10}=6$ and 20 m/s, and Figure 12b shows their counterparts of Weibull distributions. It is observed that both the K and Weibull distributions can well characterize the PDFs of simulated NRCS data.

**Figure 13:**
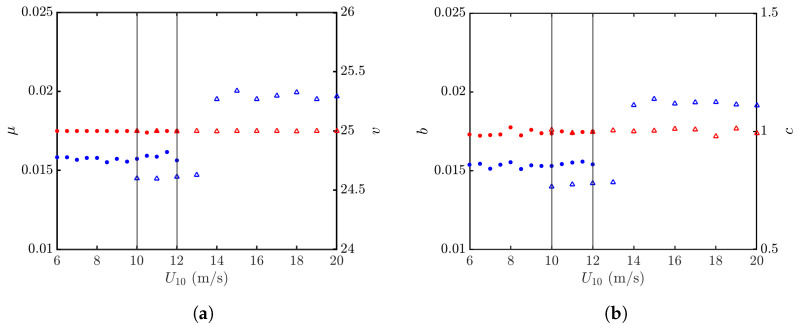
Statistical parameters versus wind speed, vv polarization. •, •: Simulated with JONSWAP spectrum; Δ, Δ: simulated with Hwang spectrum. (**a**) Regressed with K distribution, μ (•, Δ), *v* (•, Δ); (**b**) regressed with Weibull distribution *b* (•, Δ), *c* (•, Δ). The vertical grey lines indicate that simulation with Hwang spectrum starts at $U_{10}=10$ m/s, and simulation with JONSWAP spectrum ends at $U_{10}=12$ m/s.

Figure 13a shows the statistical parameters μ and *v* of the K distribution over wind speeds of $U_{10}=6$–20 m/s, and Figure 13b shows the statistical parameters *b* and *c* of the Weibull distribution over the same wind-speed range. It is observed that *v* and *c* are insensitive to the wind speed. On the other hand, μ and *b* manifest a discontinuity at $U_{10}=14$ m/s, which is attributed to different azimuthal patterns adopted in the Hwang spectrum. Note that $\Theta_{J}\left(\phi \right)$ and $\Theta_{H}\left(k_{w},\phi \right)$ are adopted in $10\leq U_{10}<14$ m/s and $14\leq U_{10}\leq 20$ m/s, respectively, in order to comply with the significant wave heights (SWHs) suggested in the Douglas sea-state table. The azimuthal pattern $\Theta_{J}\left(\phi \right)$ in (10) is independent of wind speed, whereas $\Theta_{H}\left(k_{w},\phi \right)$ in (15) is affected by the air friction velocity $u_{*}$, which in turn is a function of wind speed.

**Figure 14:**
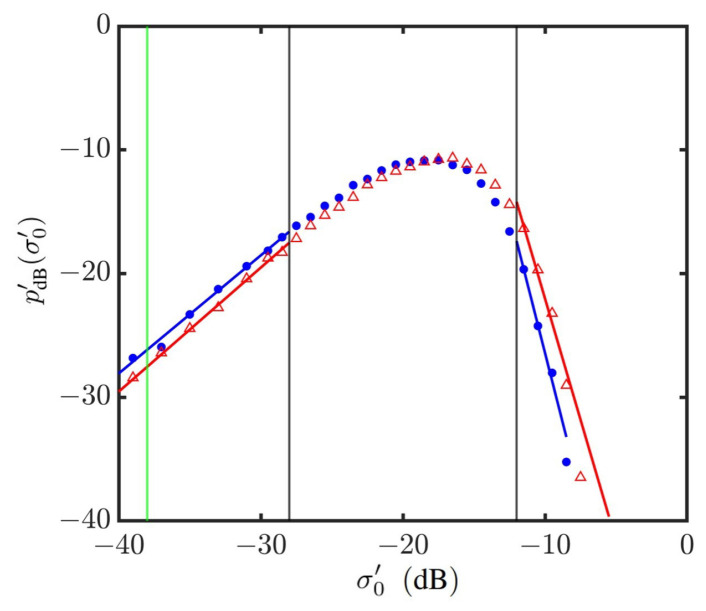
Weighted linear regression on simulated NRCS at vv polarization in outlier regions. •: Simulated with JONSWAP spectrum, $U_{10}=6$ m/s; Δ: simulated with Hwang spectrum, $U_{10}=20$ m/s. **—**: Regressed on •; **—**: regressed on Δ; **—**: noise floor at $\sigma_{0}^{\prime }=-38$ dB. The vertical grey lines indicate that $\sigma_{0}^{\prime }=-28$ dB is threshold of weak signals, and $\sigma_{0}^{\prime }=-12$ dB is threshold of strong signals.

Figure 14 shows the PDFs of NRCSs on a log–log scale, which manifests the power-law distribution in the outlier regions of strong signals and weak signals, respectively. The power-law index is estimated by applying the weighted linear regression method, with the thresholds of weak and strong signals arbitrarily set at $d_{w}=-28$ dB and $d_{s}=-12$ dB, respectively. The vertical green line marks the noise floor of typical radar systems [69].

**Figure 15:**
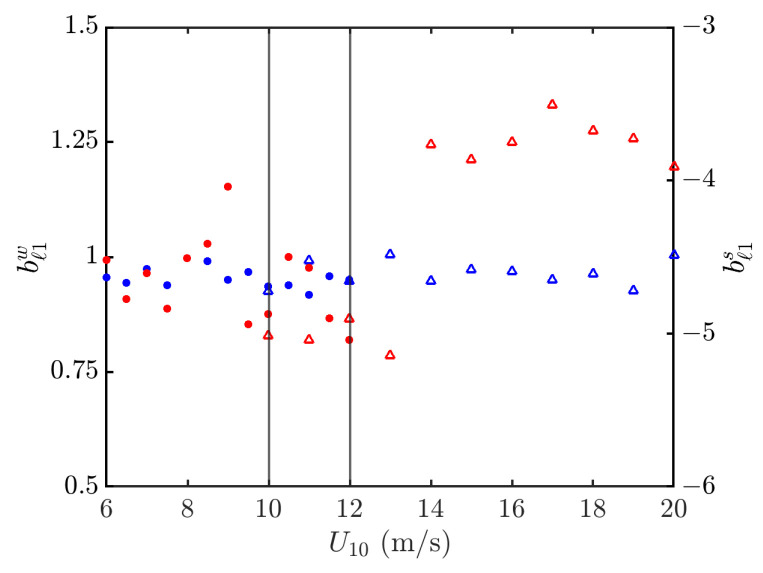
Power-law indices of PDF in outlier regions versus wind speed, vv polarization. •, •: Simulated with JONSWAP spectrum; Δ, Δ: simulated with Hwang spectrum; $b_{\ell 1}^{w}$ (•, Δ), $b_{\ell 1}^{s}$ (•, Δ). The vertical grey lines indicate that simulation with Hwang spectrum starts at $U_{10}=10$ m/s, and simulation with JONSWAP spectrum ends at $U_{10}=12$ m/s.

Figure 15 shows the power-law indices of PDFs in the outlier regions versus wind speed. It is observed that the index in the strong signal outlier region is more sensitive to the wind speed than that in the weak signal one. A discontinuity of $b_{\ell 1}^{s}$ is also manifested at about $U_{10}=14$ m/s.

### 5.2. Effects of Polarization

**Figure 16:**
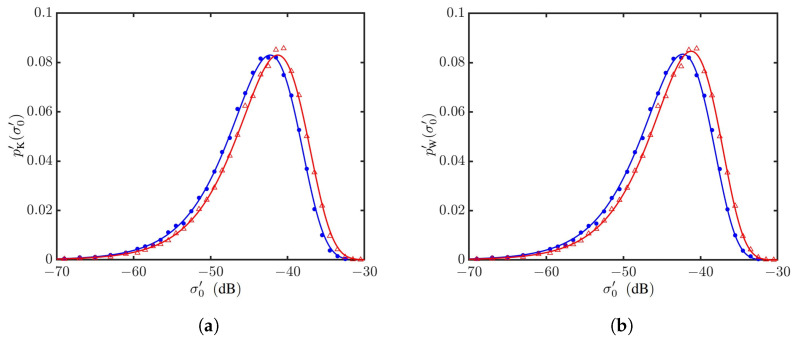
PDFs of NRCSs at hh polarization, with default parameters listed in Table 1 and Table 6. •: Simulated with JONSWAP spectrum, $U_{10}=6$ m/s; Δ: simulated with Hwang spectrum, $U_{10}=20$ m/s. **—**: Regressed on •; **—**: regressed on Δ. (**a**) Regressed with K distribution; (**b**) regressed with Weibull distribution.

Similar to vv polarization, Figure 16 shows that the PDFs of NRCSs at hh polarization can be well characterized with the K and Weibull distributions. The magnitude of $\sigma_{0vv}^{\prime }$ is larger than that of $\sigma_{0hh}^{\prime }$, which in turn is larger than those of $\sigma_{0vh}^{\prime }$ and $\sigma_{0hv}^{\prime }$.

**Figure 17:**
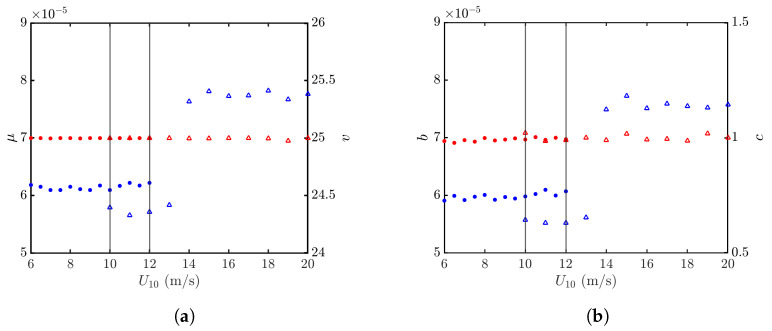
Statistical parameters versus wind speed, hh polarization. •, •: Simulated with JONSWAP spectrum; Δ, Δ: simulated with Hwang spectrum. (**a**) Regressed with K distribution, μ (•, Δ), *v* (•, Δ); (**b**) regressed with Weibull distribution, *b* (•, Δ), *c* (•, Δ). The vertical grey lines indicate that simulation with Hwang spectrum starts at $U_{10}=10$ m/s, and simulation with JONSWAP spectrum ends at $U_{10}=12$ m/s.

Figure 17 shows that the parameter *v* of the K distribution and the parameter *c* of the Weibull distribution are insensitive to the wind speed. The parameter μ of the K distribution and the parameter *b* of the Weibull distribution are more easily affected by the wind speed, and manifest a discontinuity at $U_{10}=14$ m/s, across which different azimuthal patterns are adopted.

In Figure 13 and Figure 17, the shape parameter *v* of the K distribution remains about 25 when the wind speed $U_{10}$ reaches 20 m/s (corresponding to sea state 7), implying the clutter amplitude still follows a Rayleigh distribution. By taking a second inspection of the sea-surface profile shown in Figure 4a, simulated under $U_{10}=6$ m/s, numerous potential scatterers are observed. The magnitude of the backscattering field contributed by these presumably uncorrelated scatterers is expected to follow a Rayleigh distribution. On the other hand, the sea-surface profile shown in Figure 7b, simulated under $U_{10}=20$ m/s, manifests significant long-wave features superposed with roughness of short wavelengths. Despite the long-wave features, the random sea-surface roughness presents numerous uncorrelated scatterers which contribute to the backscattering field that follows a Rayleigh distribution.

**Figure 18:**
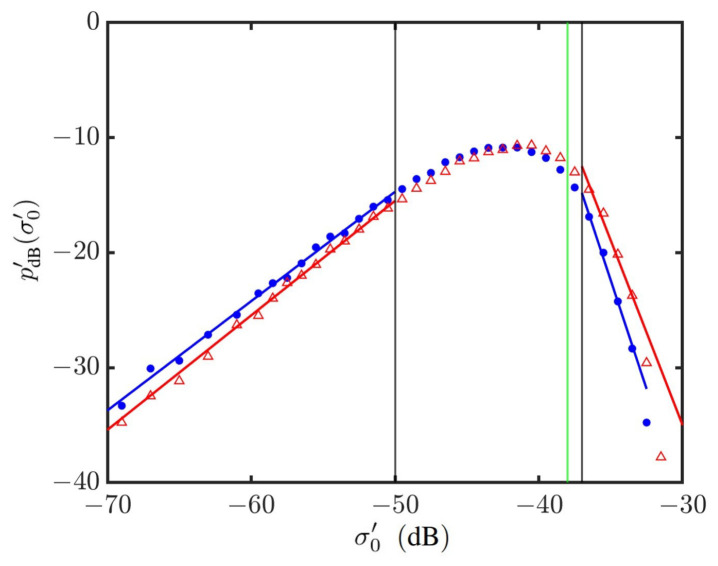
Weighted linear regression on simulated NRCSs at hh polarization in outlier regions. •: Simulated with JONSWAP spectrum, $U_{10}=6$ m/s; Δ: simulated with Hwang spectrum, $U_{10}=20$ m/s. **—**: Regressed on •; **—**: regressed on Δ; **—**: noise floor at $\sigma_{0}^{\prime }=-38$ dB. The vertical grey lines indicate that $\sigma_{0}^{\prime }=-50$ dB is threshold of weak signals, and $\sigma_{0}^{\prime }=-37$ dB is threshold of strong signals.

**Figure 19:**
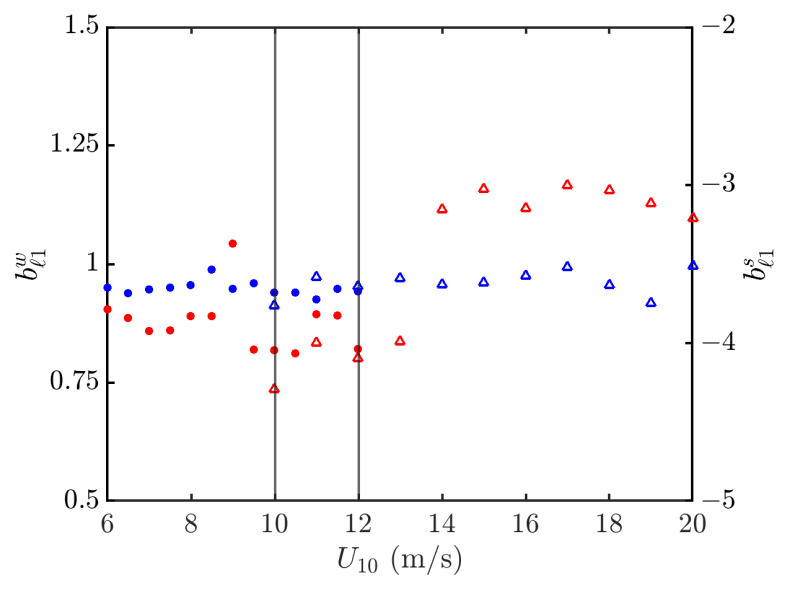
Power-law indices of PDFs in outlier regions versus wind speed, hh polarization. •, •: Simulated with JONSWAP spectrum; Δ, Δ: simulated with Hwang spectrum; $b_{\ell 1}^{w}$ (•, Δ), $b_{\ell 1}^{s}$ (•, Δ). The vertical grey lines indicate that simulation with Hwang spectrum starts at $U_{10}=10$ m/s, and simulation with JONSWAP spectrum ends at $U_{10}=12$ m/s.

Figure 18 shows the weighted linear regression on simulated NRCSs at hh polarization in the outlier regions. The outlier region of weak signals is not regressed in practice because the threshold of $d_{w}=-50$ dB is below the noise floor of $\sigma_{0}^{\prime }=-38$ dB. The threshold of strong signals is set at $d_{s}=-37$ dB, and Figure 19 shows that the power-law index varies between −4 and −3 as the wind speed varies between 6 and 20 m/s.

### 5.3. Effects of Grazing Angle

Low-earth orbit (LEO) satellites are becoming practical and viable platforms to carry radars for sensing sea surfaces. Sea clutter will still be an important factor to consider, as in conventional shipborne or coast-based radars, except large grazing angles will be engaged, pending on applications.

**Figure 20:**
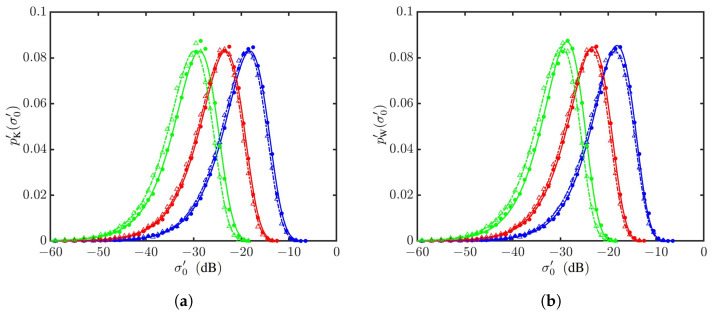
PDFs of NRCSs with default parameters listed in Table 1 and Table 6, vv polarization, $U_{10}=10$ m/s. •: Simulated with JONSWAP spectrum at $\theta_{g}=15^{\circ }$; Δ: simulated with Hwang spectrum at $\theta_{g}=15^{\circ }$; •: simulated with JONSWAP spectrum at $\theta_{g}=20^{\circ }$; Δ: simulated with Hwang spectrum at $\theta_{g}=20^{\circ }$; •: simulated with JONSWAP spectrum at $\theta_{g}=45^{\circ }$; Δ: simulated with Hwang spectrum at $\theta_{g}=45^{\circ }$. **—**: Regressed on •; **—**: regressed on •; **—**: regressed on •; **- - - - -**: regressed on Δ; **- - - - -**: regressed on Δ; **- - - - -**: regressed on Δ. (**a**) Regressed with K distribution; (**b**) regressed with Weibull distribution.

Figure 20 shows the PDFs of NRCSs with vv polarization for $U_{10}=10$ m/s and $\theta_{g}=15,20,45^{\circ }$. The PDFs drift leftwards as the grazing angle increases from $\theta_{g}=15^{\circ }$ to $45^{\circ }$. The PDFs simulated with the Hwang spectrum drift slightly leftwards from their counterparts simulated with the JONSWAP spectrum. The Weibull distribution fits better near the peak of PDFs than the K distribution.

**Figure 21:**
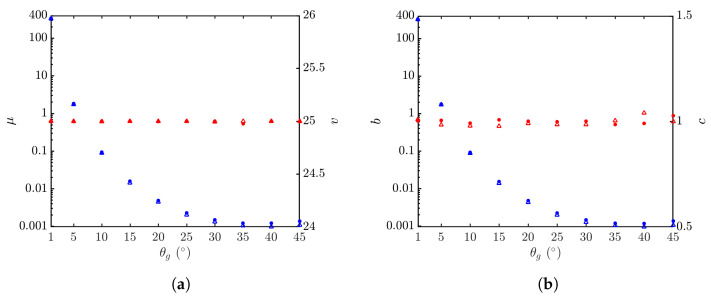
Statistical parameters versus grazing angle, with default parameters listed in Table 1 and Table 6, vv polarization, $U_{10}=10$ m/s. •, •: Simulated with JONSWAP spectrum; Δ, Δ: simulated with Hwang spectrum. (**a**) Regressed with K distribution, μ (•, Δ), *v* (•, Δ); (**b**) regressed with Weibull distribution, *b* (•, Δ), *c* (•, Δ).

Figure 21 shows the statistical parameters versus grazing angle. It is observed that the parameter *v* of the K distribution and the parameter *c* of the Weibull distribution are insensitive to the grazing angle, whereas the parameter μ of the K distribution and the parameter *b* of the Weibull distribution decrease monotonically with the grazing angle. The decreasing rate is more conspicuous at low grazing angles.

**Figure 22:**
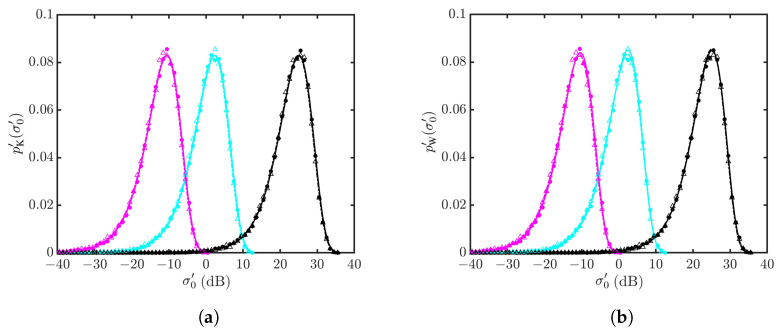
PDFs of NRCSs with default parameters listed in Table 1 and Table 6, vv polarization, $U_{10}=10$ m/s. •: Simulated with JONSWAP spectrum at Δ: simulated with Hwang spectrum at $\theta_{g}=10^{\circ }$; •: simulated with JONSWAP spectrum at $\theta_{g}=5^{\circ }$; Δ: simulated with Hwang spectrum at $\theta_{g}=5^{\circ }$; •: simulated with JONSWAP spectrum at $\theta_{g}=1^{\circ }$; Δ: simulated with Hwang spectrum at $\theta_{g}=1^{\circ }$. **—**: Regressed on •; **—**: regressed on •; **—**: regressed on •; **- - - - -**: regressed on Δ; **- - - - -**: regressed on Δ; **- - - - -**: regressed on Δ. (**a**) Regressed with K distribution; (**b**) regressed with Weibull distribution.

Figure 22 shows the PDFs of NRCSs at $\theta_{g}=1^{\circ },5^{\circ }$, and $10^{\circ }$. Their shapes are very close to one another, their *v* parameters are close to 25 and their *c* parameters are close to one, as shown in Figure 21. As $\theta_{g}$ is decreased from $10^{\circ }$ to $1^{\circ }$, the NRCS value (backscattering signal strength) increases monotonically, accompanied by the monotonical increase in parameters μ and *b*.

The empirical model in [17] for estimating the shape parameter *v* of the K distribution at low grazing angles was based on measurement data, which were inevitably affected by many uncertainties. In our framework, the simulation parameters listed in Table 1 and Table 6 bear no uncertainties, implying that the PDFs of simulated NRCSs can be attributed to specific wind conditions (sea states). The results presented in Figure 21 are obtained under $U_{10}=10$ m/s, corresponding to a medium-to-high sea state or Douglas sea state 5.

The shape parameter in [17] varied with grazing angle at low sea state and low grazing angles. The variation in the shape parameter at sea state 6 and $\theta_{g}>1^{\circ }$ was limited. The shape parameter at vv polarization in $15^{\circ }\leq \theta_{g}\leq 45^{\circ }$ varied without trend, partially attributed to the uncertainties embedded in the measurement data.

### 5.4. Effects of Wind Direction

**Figure 23:**
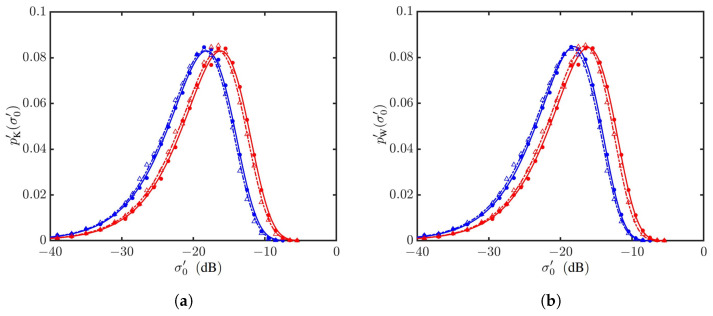
PDFs of NRCSs, with default parameters listed in Table 1 and Table 6, vv polarization, $U_{10}=10$ m/s. •: Simulated with JONSWAP spectrum at $\phi_{wd}=0^{\circ }$; Δ: simulated with Hwang spectrum at $\phi_{wd}=0^{\circ }$; •: simulated with JONSWAP spectrum at $\phi_{wd}=90^{\circ }$; Δ: simulated with Hwang spectrum at $\phi_{wd}=90^{\circ }$. **—**: Regressed on •; **—**: regressed on •; **- - - - -**: regressed on Δ; **- - - - -**: regressed on Δ. (**a**) Regressed with K distribution; (**b**) regressed with Weibull distribution.

Figure 23 shows PDFs of NRCSs with vv polarization, $U_{10}=10$ m/s, and $\phi_{wd}=0$ and $90^{\circ }$. It is observed that the mean value of NRCSs at $\phi_{wd}=90^{\circ }$ is larger than that at $\phi_{wd}=0^{\circ }$. The mean value simulated with the JONSWAP spectrum is slightly larger than its counterpart simulated with the Hwang spectrum.

**Figure 24:**
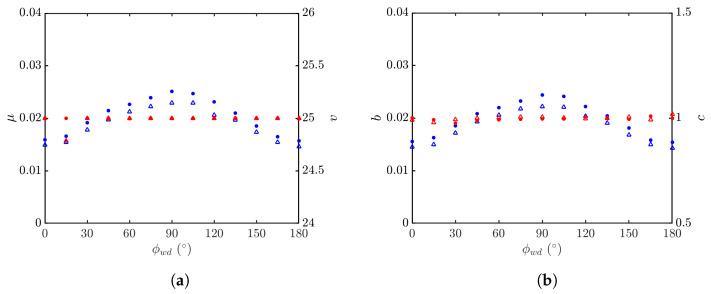
Statistical parameters versus wind direction, with default parameters listed in Table 1 and Table 6, vv polarization, $U_{10}=10$ m/s. •, •: Simulated with JONSWAP spectrum; Δ, Δ: simulated with Hwang spectrum. (**a**) Regressed with K distribution, μ (•, Δ), *v* (•, Δ); (**b**) regressed with Weibull distribution, *b* (•, Δ), *c* (•, Δ).

Figure 24 shows the statistical parameters versus wind direction. It is observed that the parameter *v* of the K distribution and the parameter *c* of the Weibull distribution are insensitive to the wind direction. The parameter μ of the K distribution and the parameter *b* of the Weibull distribution monotonically increase as $\phi_{wd}$ sweeps from 0 to $90^{\circ }$, and monotonically decrease as $\phi_{wd}$ sweeps from 90 to $180^{\circ }$, in an almost symmetrical pattern about $\phi_{wd}=90^{\circ }$. The magnitudes of μ and *b* simulated with the JONSWAP spectrum are larger than their counterparts simulated with the Hwang spectrum.

**Figure 25:**
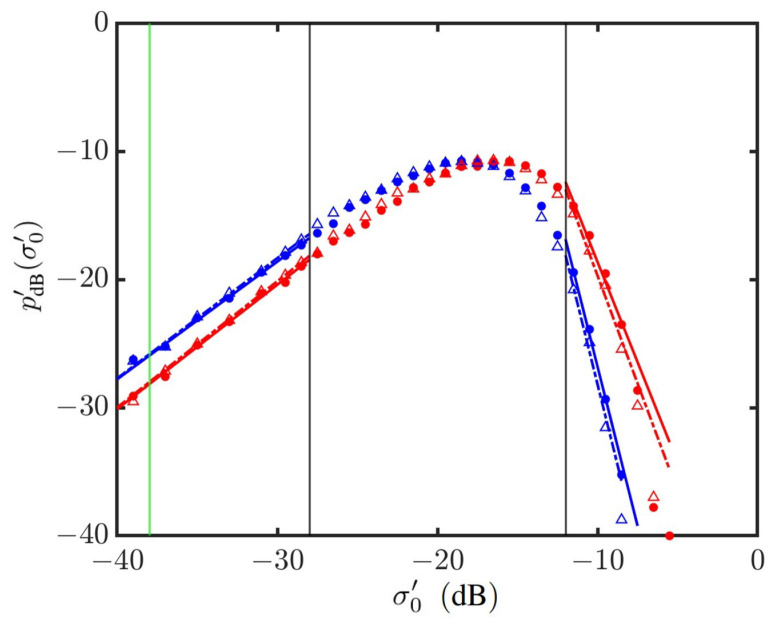
Weighted linear regression on simulated NRCSs in outlier regions, vv polarization. •: Simulated with JONSWAP spectrum at $\phi_{wd}=0^{\circ }$; Δ: simulated with Hwang spectrum at $\phi_{wd}=0^{\circ }$; •: simulated with JONSWAP spectrum at $\phi_{wd}=90^{\circ }$; Δ:simulated with Hwang spectrum at $\phi_{wd}=90^{\circ }$. **—**: Regressed on •; **—**: regressed on •; **- - - - -**: regressed on Δ; **- - - - -**: regressed on Δ; **—**: noise floor at $\sigma_{0}^{\prime }=-38$ dB. The vertical grey lines indicate that $\sigma_{0}^{\prime }=-28$ dB is threshold of weak signals, and $\sigma_{0}^{\prime }=-12$ dB is threshold of strong signals.

**Figure 26:**
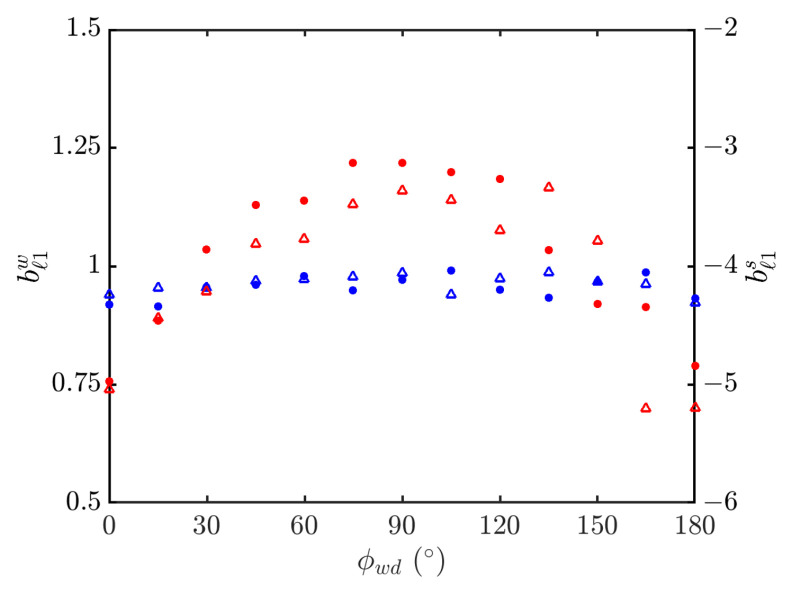
Power-law indices of PDFs in outlier regions versus wind direction, vv polarization, •, •: simulated with JONSWAP spectrum; Δ, Δ: simulated with Hwang spectrum; $b_{\ell 1}^{w}$ (•, Δ), $b_{\ell 1}^{s}$ (•, Δ).

Figure 25 shows the weighted linear regression on simulated NRCSs in the outlier regions, with $\phi_{wd}=0$ and $90^{\circ }$. Figure 26 shows the power-law indices of PDFs in the outlier regions versus wind direction. It is observed that the index in the outlier region of weak signals is insensitive to the wind direction. The index in the outlier region of strong signals increases monotonically from −5 at $\phi_{wd}=0$ to −3 at $\phi_{wd}=90^{\circ }$, and decreases monotonically to −5 at $\phi_{wd}=180^{\circ }$, in an almost symmetrical pattern about $\phi_{wd}=90^{\circ }$.

### 5.5. Highlights and Prospects

Collecting field measurement data on sea clutter may be expensive to cover all possible radar operation conditions, under conceivable combinations of wind speed, wind direction, polarization, and grazing angle, among others. A complete framework of predicting the characteristics of sea clutter under specific radar operation conditions will be useful to a wide variety of applications, and can be used as a reference or guidelines for designing future measurement tasks to enhance the existing empirical models on ocean-wave spectra, normalized radar cross-sections (NRCSs), and so on. The proposed framework is composed of empirical spectra used to characterize sea-surface profiles under different wind speeds, the Monte Carlo method to generate realizations of sea-surface profile, the physical-optics method to compute the NRCSs from a multitude of sea-surface realizations, and regression of NRCS data (sea clutter) with empirical probability density functions (PDFs) to derive a few statistical parameters.

An ensemble of sea-surface profiles under wind speeds of $U_{10}=6$–20 m/s are realized by applying the Monte Carlo method upon the JONSWAP spectrum at low-to-medium wind speeds and the Hwang spectrum at high wind speeds. These two spectra are adopted for demonstration. More robust ocean-wave spectra can be developed and their efficacy can be evaluated by comparing the realizations of sea-surface profiles with observed ones.

The physical-optics method is used to compute the NRCS from an individual sea-surface realization, which is approximated by a set of triangular patches. This method works well on one premise, that the patch size is at least a few radar wavelengths. Thus, sea-surface ripples with wavelengths of cm or less cannot be perceived if X-band radar is used. More sophisticated electromagnetic models can be developed to compute the NRCS, possibly at higher computational cost.

The PDFs of NRCSs, under different operational conditions, are well regressed with the K or Weibull distributions, each characterized by two statistical parameters, plus two power-law indices for characterizing weak and strong signals. The power-law distributions for weak and strong radar echoes were not found in the literature. The dependence of these statistical parameters and power-law indices upon wind speed, wind direction, polarization, and grazing angle is explored, which can be used to quickly predict these statistical parameters and power-law indices when a specific operational condition is given. These statistical parameters and indices can be used to reconstruct the PDFs of NRCSs effectively and efficiently for predicting the properties of sea clutter under given wind and radar operation conditions.

Sea-clutter-related issues have been widely discussed in the literature. Recently, three adaptive detectors were proposed to detect a target immersed in a sea-clutter dominant scenario [70]. An asymmetric adaptive detection problem was solved, with sea clutter characterized by a compound-Gaussian distribution with inverse-Gaussian (CG-IG) texture. Statistical parameters were estimated by applying a moment estimator and a Nelder–Mead algorithm. The CG-IG distribution was validated with the South Africa Fynmeet sea clutter dataset in a specific scenario. In comparison, our framework provides more flexibility and can be applied to describe more diverse sea-clutter distributions under different sea states and radar conditions, which will benefit the development of target detection methods.

In [71], co-polarized and cross-polarized bistatic coherent sea-clutter returns were investigated with statistical inference. A spherically invariant random process (SIRP) was applied to describe the statistical properties of sea clutter, assuming a wide-sense stationary texture and speckle of sea clutter. Our framework can deal with more versatile sea states and radar conditions in the simulations, including wind speed, wind direction, grazing angle, and polarization.

In [72], a distribution of sea clutter was regressed with a statistical model composed of the gamma distribution and its second moment to facilitate ship detection. It was validated with measured sea-clutter data under low-to-medium wind speeds at L-band. In comparison, our framework has been validated to cover wind speeds up to 20 m/s.

In [73], the statistical properties of sea clutter, under low grazing angles, were modeled with the compound K distribution and gamma-distributed texture, and were studied via two examples of monochromatic swell pattern and simulated sea-surface profiles with empirical ocean-wave spectra, respectively. Separate memoryless nonlinear transformations (MNLTs) were applied on the simulated sea-surface profiles to acquire the characteristics of the Doppler spectrum. It was reported that further studies on the relationship between the empirical parameters in the model and the environmental conditions were needed, which can be implemented by applying our framework.

In [74], S-band sea clutter from a NetRAD radar system, at low grazing angles, was analyzed with a Suzuki distribution, which was a compound Gaussian model with log-normal texture. It was shown that the Suzuki distribution could be transformed to obtain the normally distributed texture directly related to the sea-surface slope. The water depth and wave direction were estimated by using a dispersion relation derived from the two-dimensional range-time autocorrelation function (ACF) of sea clutter. Our framework can account for more versatile sea states and radar conditions in the simulations, providing useful clues for similar studies like this.

In [75], a two-dimensional amplitude and phase matching optimization (APMO) method was proposed to simulate spatial–temporal correlated sea clutter in two steps. First, a frequency-domain inverse transform and a correlation transfer were applied on the measured sea clutter from NAU clutter data to simulate clutter amplitudes with similar distribution. Then, the clutter phases were estimated by applying an optimization method. In our framework, the sea clutter is computed from sea-surface realizations simulated in terms of an empirical ocean-wave spectrum, which can be flexibly adjusted to simulate a wide variety of sea states.

Note that the third part of the proposed framework can be applied to the PDF derived from measurement NRCS data, $\sigma_{0}^{\mathrm{obs}}$. Imagine a framework comparable to that shown in the flowchart of Figure 1 was conducted by measurement, the cost spent in acquiring the measurement data to derive PDF $p^{\prime }\left(\sigma_{0}^{\mathrm{obs}}\right)$, over the same ranges of wind conditions and radar parameters as presented in Section 5, would be much higher than the cost of simulations.

## 6. Conclusions

A complete framework of predicting the properties of sea clutter, under different operational conditions specified by wind speed, wind direction, grazing angle, and polarization, is proposed for the first time. This framework is composed of empirical spectra used to characterize sea-surface profiles under different wind speeds, the Monte Carlo method to generate realizations of sea-surface profiles, the physical-optics method to compute the normalized radar cross-sections (NRCSs) from a multitude of sea-surface realizations, regression of NRCS data (sea clutter) with empirical probability density functions (PDFs) to derive a few statistical parameters, and power-law indices via a particle swarm optimization (PSO) algorithm. The JONSWAP and Hwang spectra of ocean waves are adopted to generate realizations of sea-surface profiles at low and high wind speeds, respectively. The probability density functions of NRCSs are regressed with the K and Weibull distributions, each characterized by two parameters. The probability density functions in the outlier regions of weak signals and strong signals are regressed with power laws, each characterized by an index. These statistical parameters of the K and Weibull distributions, including the power-law indices, are investigated for the first time under different operational conditions. The study reveals useful and succinct information of sea clutter that can be used to improve the radar performance in a wide variety of complicated ocean environments. The proposed framework can be used as a reference or guidelines for designing future measurement tasks to enhance existing empirical models.

## Figures and Tables

**Table 1:** Default parameters for simulating sea-surface profiles with JONSWAP or Hwang spectra.

Parameter	Symbol	Value
Observation period	$T_{ob}$	10 s
Sampling frequency	$f_{a}$	100 Hz
Surface resolution	Δx,Δy	4.9087 m
Wavenumber resolution	$\Delta k_{wx}$, $\Delta k_{wy}$	0.01 rad/m
*x* length	$L_{x}$	628.3185 m
*y* length	$L_{y}$	628.3185 m
Illuminating area	*A*	394,784.176 m^2^
Number of samples in *x* dir.	$N_{x}$	128
Number of samples in *y* dir.	$N_{y}$	128
Wind speed	$U_{10}$	10 m/s
Fetch length	*F*	50 m
Parameter $\sigma_{a}$	$\sigma_{a}$	0.06 [48]
Parameter $\sigma_{b}$	$\sigma_{b}$	0.1 [48]
Peak enhancement factor	γ	3.3 [49]
Wind direction	$\phi_{wd}$	$180^{\circ }$
Empirical phase coefficient	$\alpha_{c}$	128
Ratio of surface pressure to water mass density	$\gamma_{r}$	$7.1585\times 10^{-5}$ m^3^/s^2^ [51]

**Table 2:** Maximum amplitudes of JONSWAP spectra.

$U_{10}$ (m/s)	$|H_{J}|$ (dB)
6	66.5116
8	76.7065
10	85.1549
12	91.1055

**Table 3:** Significant wave height (SWH) simulated with JONSWAP spectrum.

$U_{10}$	Simulated SWH	SWH in [52]
6 m/s	0.7921 m	0.3–0.9 m
8 m/s	1.5600 m	1.5–2.4 m
10 m/s	2.5877 m	1.5–2.4 m
12 m/s	3.8939 m	2.4–3.7 m

**Table 4:** Maximum amplitude of Hwang spectra.

$U_{10}$ (m/s)	$|H_{H}|$ (dB)
10	90.6582
12	91.6060
16	96.1053
20	97.2381

**Table 5:** Significant wave height (SWH) simulated with Hwang spectrum.

$U_{10}$	Simulated SWH	SWH in [52]
10 m/s	2.1756 m	1.5–2.4 m
12 m/s	2.4264 m	2.4–3.7 m
16 m/s	5.8004 m	6.1–12.2 m
20 m/s	6.5855 m	6.1–12.2 m

**Table 6:** Default radar parameters.

Parameter	Symbol	Value
Radar center frequency	$f_{0}$	10.1 GHz [32]
Slant range	$R_{0}$	15.4169 km
Grazing angle	$\theta_{g}$	$15^{\circ }$
Polarization	vv	
3 dB azimuth beamwidth	$\phi_{a}$	$1^{\circ }$ [68]
3 dB elevation beamwidth	$\theta_{e}$	$13^{\circ }$ [68]

## Data Availability

Publicly available datasets were analyzed in this study.

## Appendix A. Comparison of Pierson–Moskowitz, Mitsuyasu, and Elfouhaily Spectra

The Pierson–Moskowitz (PM) spectrum is given by [6]

$$\begin{array}{ccc} & & \;\Phi_{\mathrm{PM}}\left(\omega_{w}\right)=\frac{\alpha g^{2}}{\omega_{w}^{5}}e^{-\beta {\left(\omega_{\max}/\omega_{w}\right)}^{4}} \end{array}$$

where $\alpha =8.1\times 10^{-3}$ , β=0.74, and $\omega_{\max}$ is the frequency of the spectral peak. The PM spectrum was proposed by modifying the Phillips’ spectrum of $\Phi_{\mathrm{Ph}}\left(\omega_{w}\right)=\alpha g^{2}/\omega_{w}^{5}$ [76], to characterize fully developed wind-driven sea surfaces, featuring gravity waves [7]. On the other hand, the PM spectrum is not suitable for characterizing developing sea surfaces.

The Mitsuyasu spectrum $\Phi_{M}\left(\omega_{w}\right)$ was proposed by modifying the coefficients of the Phillips’ spectrum with field measurements of limited fetch length *F* in a wave tank and in Hakata Bay. Its spectrum is given by [77]

$$\begin{array}{ccc} & & \;\Phi_{M}\left(\omega_{w}\right)=\frac{g^{2}}{\omega_{w}^{5}}{\left(21\log_{10}\frac{gF}{u_{*}^{2}}-34.5\right)}^{-1} \end{array}$$

if $\omega_{w}\geq \omega_{\max}$, and

$$\begin{array}{ccc} & & \;\Phi_{M}\left(\omega_{w}\right)=1.66\times 10^{-9}\frac{g^{2}}{\omega_{w}^{5}}\exp\left\{3.94{\left(\frac{gF}{u_{*}^{2}}\right)}^{0.283}\frac{u_{*}\omega_{w}}{g}\right\} \end{array}$$

if $\omega_{w}\leq \omega_{\max}$, where $u_{*}$ is the surface friction velocity. Similar to the PM spectrum, the Mitsuyasu spectrum applies to fully developed wind-driven sea surfaces [78].

The JONSWAP spectrum $\Phi_{J}\left(\omega_{w}\right)$ in (7) was extended from the PM spectrum to cover both developing and fully developed sea surfaces [48]. Its functional form was determined by five parameters ($\omega_{\max}$, α, γ, $\sigma_{a}$, and $\sigma_{b}$) via regressing with the spectra observed under different ocean-wave generation conditions [48]. The peak of the JONSWAP spectrum is equal to that of the PM spectrum multiplied by the base of peak enhancement factor, γ. The conventional JONSWAP spectrum is characterized by γ=3.3, $\sigma_{a}=0.07$, and $\sigma_{b}=0.09$, which are not strictly tenable [48]. It was claimed in [49] that γ=1 characterizes fully developed sea surfaces, with the JONSWAP spectrum reduced to the PM spectrum; and γ>1 characterizes developing sea surfaces. In this work, we choose the values of γ=3.3, $\sigma_{a}=0.06$, and $\sigma_{b}=0.1$ to characterize developing sea surfaces.

The Elfouhaily spectrum was proposed by modifying the JONSWAP spectrum, as [51]

$$\begin{array}{ccc} & & \;\Phi_{\mathrm{El}}\left(k_{w}\right)=\frac{2\pi }{k_{w}^{3}}\left[B_{\ell }\left(k_{w}\right)+B_{h}\left(k_{w}\right)\right] \end{array}$$

where $B_{\ell }\left(k_{w}\right)$ and $B_{h}\left(k_{w}\right)$ are the spectra at low and high wavenumbers, respectively, with the explicit forms

$$\begin{array}{ccc} & & \;B_{\ell }\left(k_{w}\right)=\frac{1}{2}\alpha_{p}\frac{V_{p}}{V_{w}}F_{p}\left(k_{w}\right)\\ & & \;B_{h}\left(k_{w}\right)=\frac{1}{2}\alpha_{m}\frac{V_{\min}}{V_{w}}F_{m}\left(k_{w}\right) \end{array}$$

$V_{p}=V_{w}\left(k_{p}\right)$ is the phase speed at the spectral peak $k_{p}$, and $\alpha_{p}$ and $\alpha_{m}$ are the generalized Phillips–Kitaiporodskii equilibrium range parameters at low and high wavenumbers, respectively [51].

The explicit form of the side-effect function $F_{p}\left(k_{w}\right)$ at low wavenumbers is modified from its counterpart in the JONSWAP spectrum as

$$\begin{array}{ccc} & & \;F_{p}\left(k_{w}\right)=e^{-1.25{\left(\omega_{\max}/\omega_{w}\right)}^{4}}\gamma^{\exp\{-{\left(\omega_{w}-\omega_{\max}\right)}^{2}/\left(2\sigma^{2}\omega_{\max}^{2}\right)\}}\\ & & \exp\left\{-\frac{\Omega }{\sqrt{10}}\left(\sqrt{\frac{k_{w}}{k_{p}}}-1\right)\right\} \end{array}$$

where Ω is the inverse wave-age parameter, and the third term on the right-hand side accounts for the observed spectral roll-off in tank experiments [79]. Similarly, the explicit form of $F_{m}\left(k_{w}\right)$ is given by

$$\begin{array}{ccc} & & \;F_{m}\left(k_{w}\right)=e^{-1.25{\left(\omega_{\max}/\omega_{w}\right)}^{4}}\gamma^{\exp\{-{\left(\omega_{w}-\omega_{\max}\right)}^{2}/\left(2\sigma^{2}\omega_{\max}^{2}\right)\}}\\ & & \exp\left\{-\frac{1}{4}{\left(\frac{k_{w}}{k_{\min}}-1\right)}^{2}\right\} \end{array}$$

where the third term on the right-hand side accounts for the viscous cutoff of gravity-capillary waves [51].

The Elfouhaily spectrum reveals more precise properties of gravity-capillary and capillary waves in the high-wavenumber regime. It is suitable to describe finer ocean-wave features at low sea states under low wind speeds. However, its difference from the JONSWAP spectrum is indiscernible at moderate wind speeds.
